# Recent advances in prevention, detection and treatment in prosthetic joint infections of bioactive materials

**DOI:** 10.3389/fbioe.2022.1053399

**Published:** 2022-11-10

**Authors:** Hongbin Xie, Yubo Liu, Haoming An, Jiafeng Yi, Chao Li, Xing Wang, Wei Chai

**Affiliations:** ^1^ Senior Department of Orthopedics, Fourth Medical Center of People’s Liberation Army General Hospital, Beijing, China; ^2^ School of Medicine, Nankai University, Tianjin, China; ^3^ National Clinical Research Center for Orthopaedics, Sports Medicine & Rehabilitation, Beijing, China; ^4^ Beijing National Laboratory for Molecular Sciences, Institute of Chemistry, Chinese Academy of Sciences, Beijing, China; ^5^ University of Chinese Academy of Sciences, Beijing, China

**Keywords:** periprosthetic joint infection, bioactive materials, biomaterials, tissue engineering, joint replacement

## Abstract

Prosthetic joint infection (PJI) is often considered as one of the most common but catastrophic complications after artificial joint replacement, which can lead to surgical failure, revision, amputation and even death. It has become a worldwide problem and brings great challenges to public health systems. A small amount of microbe attaches to the graft and forms a biofilm on its surface, which lead to the PJI. The current standard methods of treating PJI have limitations, but according to recent reports, bioactive materials have potential research value as a bioactive substance that can have a wide range of applications in the field of PJI. These include the addition of bioactive materials to bone cement, the use of antibacterial and anti-fouling materials for prosthetic coatings, the use of active materials such as bioactive glasses, protamine, hydrogels for prophylaxis and detection with PH sensors and fluorescent-labelled nanoparticles, and the use of antibiotic hydrogels and targeting delivery vehicles for therapeutic purposes. This review focus on prevention, detection and treatment in joint infections with bioactive materials and provide thoughts and ideas for their future applications.

## 1 Introduction

### 1.1 Prosthetic joint infections

With the development of population aging, the incidence of lower extremity joint diseases is increasing in the elderly population, and seriously affects the quality of life of the elderly. Total joint arthroplasty (TJA) is mainly used to treat end-stage joint diseases. The surgery involves removing part or all of the damaged joint and fitting hardware—orthopaedic prostheses made of metal, plastic, ceramic or a combination of these materials (Innes and Atwater, 2020) to allow movement without pain or restriction and it can relieve joint pain, correct joint deformity, restore joint function and enhance the quality of life. Because of these advantages, TJA is widely used around the world. One of the most commonly encountered and devastating post-arthroplasty complications is prosthetic joint infection (PJI) (Rodríguez-Pardo et al., 2015). This may require revision surgery and in some severe cases, it even brings out amputation (Kapadia et al., 2016) or death (Izakovicova et al., 2019). Therefore, PJI has become a worldwide challenge and imposes a huge economic burden on public health systems (Premkumar et al., 2021).

Regarding to the classification of PJI, there is no sufficiently clear international delineation of the clinical classification of PJI. In a review by Costa-Pinto et al., (2021), it provides a rational classification of PJI based on cause, development of the infection and treatment. According to this classification, PJI is divided into three categories: early/acute, delayed/subacute and late/chronic. Early/acute PJI usually occurs less than 3 months after surgery and is commonly caused by intra-operative contamination, usually treated with debridement, antibiotic treatment and retention of the prosthesis. Delayed/subacute PJI usually occurs 3–24 months after surgery and can be acquired at the time of surgery (caused by less virulent microorganisms), mostly treated with debridement and prosthetic retention or removal. Delayed/subacute PJI usually occurs more than 2 years after surgery and is usually initially asymptomatic, mostly caused by bloodstream infection, but can also be caused by intraoperative contamination. Treatment usually includes debridement, antibiotic therapy, revision, and the prosthesis is often not reserved. Thus, the appropriate treatment can be selected according to the type of PJI and the degree of infection.

### 1.2 Mechanisms of prosthetic joint infections

**FIGURE 1:**
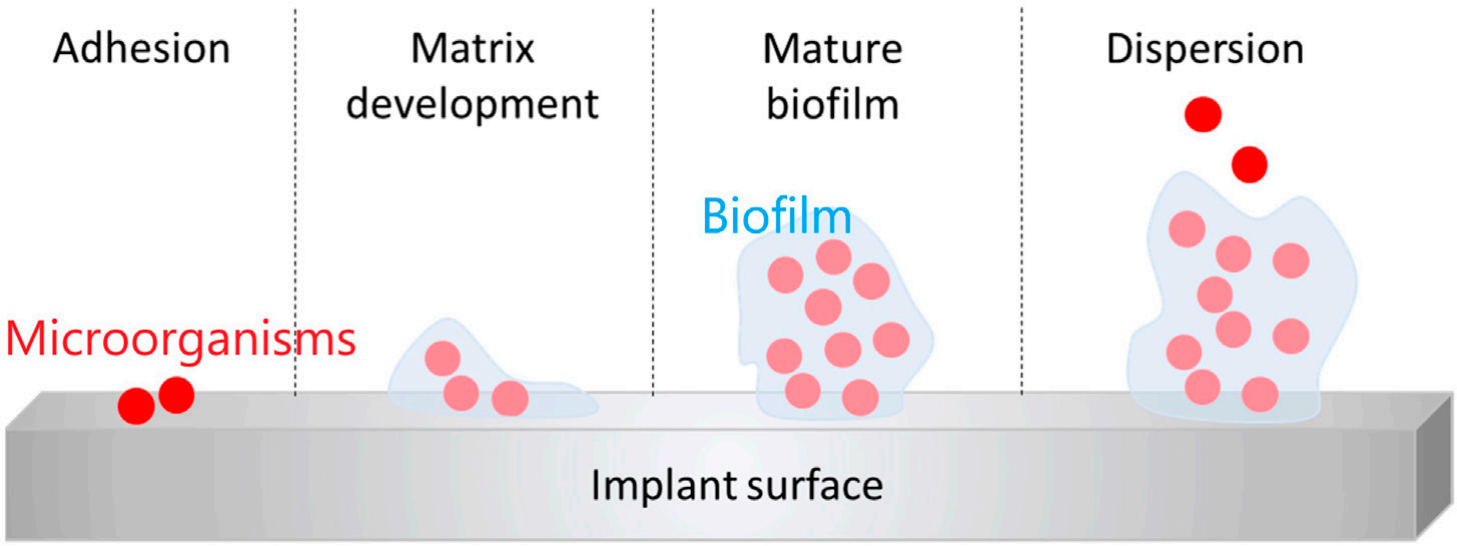
Schematic preparation of the steps of biofilm formation (Bistolfi et al., 2019)., Copyright 2019, Materials.

The main causes of PJI are microbial adhesion and biofilm formation. It accounts for 60% of hospital-associated infections (Zilberman and Elsner, 2008). Infections caused by biofilm formation may lead to tissue destruction, systemic transmission of pathogens, severe systemic disease and even death (Hall-Stoodley et al., 2004). Implant-associated infections are caused by bacterial adhesion, which is influenced by several factors: 1) hydrophobicity and surface charge of the bacteria; 2) factors on the implant surface; 3) environmental factors; 4) tissue factors in the blood; 5) specific adhesion molecules (Ribeiro et al., 2012). A biofilm is a complex and organized colony of microorganisms whose surface is a self-produced polymer matrix of polysaccharides, proteins, and DNA in a matrix. This allows biofilms to adhere to exposed surfaces (Jolivet-Gougeon and Bonnaure-Mallet, 2014). Following this, a series of events occur (Figure 1): a new cycle of cell attachment, adhesion, matrix development, mature biofilm and dispersion from the biofilm, which in turn generates biofilms elsewhere.

A variety of molecules are transported in the polysaccharide matrix in the biofilm and can provide environmental structural stability to bacteria (McConoughey et al., 2014). Biofilms are also interspersed with a variety of water channels that improve the nutrition and metabolism of microorganisms (Høiby et al., 2010). These factors make biofilms resistant to antibiotics, tolerant to disinfectants, and immune system-resistant (Arciola et al., 2015).

### 1.3 Current strategies to treat prosthetic joint infections

Standard treatments of PJI include taking out the infected joint prosthesis, eliminating the necrotic bone fragments, using antibiotics locally and/or systemically, and revising the joint with a new prosthesis after the infection has been healed thoroughly (Costa-Pinto et al., 2021). Although suffer such rigorous treatment, compared to uninfected TJA patients, patients with PJI will experience poorer quality of post-operative life (Kapadia et al., 2016) and face higher 5-year mortality rate (Berend et al., 2013). Bacterial biofilm’s capacity to withstand mechanical debridement and antibiotics is one of the primary causes of PJI and one of the reasons it is challenging to eradicate (Levack et al., 2018). So proper prevention, detection and treatment in PJI is significant.

To avoid this situation, polymethylmethacrylate (PMMA) bone cement is often used in orthopedics as the gold standard biomaterial for antibiotic treatment of surgical sites (Inzana et al., 2016). The addition of antibiotic powder to PMMA bone cement was first used and described over 60 years ago as a means of preventing PJI. Nowadays, PMMA bone cement is used as a routine medical step in joint replacements in most countries around the world (Engesæter et al., 2011). However, PMMA bone cement, the gold standard biomaterial for topical antibiotic administration, has several unavoidable drawbacks. Limited antibiotic release, compatibility issues with a variety of antibiotics, a lack of biological activity, and the requirement for surgery to remove non-biodegradable bone cement before revision surgery are some of these drawbacks (Inzana et al., 2016). Recently, bioactive materials have emerged as potentially valuable biomaterials with biodegradability, minimal immunogenicity, ability to heal wounds, antibacterial action, and anti-inflammatory potential (Costa-Pinto et al., 2021). Compared to most synthetic polymers, bioactive materials are less toxic and more biocompatible. Therefore, there is great scope for research on bioactive materials in response to PJI.

## 2 Bioactive materials in prevention of PJI

As previously mentioned, the now commonly used PMMA bone cement still has many limitations. Therefore, the prevention of PJI has been thoroughly researched using the new bioactive materials.

### 2.1 Bone cement with bioactive material

In order to reduce the deficiency of existing bone cements, bioactive materials have been added to bone cements to increase their effectiveness in many studies.

#### 2.1.1 Quaternary ammonium and alkoxysilane

In the study by Wang et al. (2021), they add γ-methacryloxypropyltrimetoxysilane to provide the biological activity of PMMA cement and use 2-(tert-butylamino) ethyl methacrylate (TBAEMA) to increase antimicrobial performance. By using quaternary ammonium salts, alkoxysilanes, and water-soluble calcium salts as chemical modifiers, PMMA cements exhibiting both bioactive and antimicrobial activities were obtained. The bone cements were tested for mechanical properties, bioactivity, antibacterial activity. According to the findings, the manufactured bone cements could adhere to ISO 5833s requirement for TBAEMA concentrations of 5% or less and exhibit antimicrobial properties against both gram-positive and gram-negative bacteria.

#### 2.1.2 Chitosan and graphene oxide

In a study by Valencia Zapata et al. (2019), they added chitosan (CS) and graphene oxide (GO) to Acrylic Bone Cements (ABC) and studied their physicochemical, thermal, mechanical and biological properties. The majority of the ABCs in use today are made up of two elements: a solid based on polymethyl methacrylate (PMMA) and a liquid based on methyl methacrylate (MMA) (Hendriks et al., 2004), which are mixed to produce a polymeric monomer reaction in a chemical reaction that then becomes a hardened cement paste (Deb, 2008).

According to Zapata (Valencia Zapata et al., 2019) group’s finding, the addition of nanosheets composed of GO increased the antimicrobial activity, roughness and bending behavior of ABC, and also gave good dispersion of ABC. These altered physical properties are caused by the specific morphology of the nanosheets. In contrast, the addition of CS increased the porosity, degradation rate but decreased the compression properties of ABCs. This is due to the higher viscosity of the slurry produced by the presence of CS, which promotes the retention of air within the cement during the mixing process. From a biological point of view, the increased roughness and porosity facilitates osteoblast adhesion, proliferation, mineralisation and protein adsorption thereby improving the cytocompatibility of the osteoblasts. As a result, better thermal stability, flexural modulus, antibacterial behavior, and osteogenic activity were demonstrated by ABCs containing both GO and CS, which offer great promise for orthopedic applications. In addition to this, all ABCs were free of cytotoxicity and supported good cell viability of human osteoblasts (HOb). The ABC nanocomposites made with 15% CS and 0.3% GO (CS + GO) were ultimately discovered to have a synergistic impact on physical, mechanical, thermal, and antibacterial characteristics after comparing the findings obtained with the various formulations. These findings imply that the formulation has a strong potential for usage as an orthopedic antimicrobial bioactive cement.

#### 2.1.3 Bioactive glass

In the current study by Wekwejt et al. (2021), it was found that although Nanosilver-loaded PMMA bone cement (BC-AgNp) had antibacterial activity, it still lacked biodegradability and bioactivity. Bioactive glasses were then used to increase the bioactivity of the bone cement. The researcher doped bioactive glasses of different particle sizes into BC-AgNp and examined the effects of bioactive glasses addition using microscopic analysis, mechanical testing, cytocompatibility and antimicrobial efficiency studies. The results showed that the bone cements incorporated with different sizes of bioactive glasses had different properties. Bioactive glasses incorporated with smaller particles (40 μm) exhibited higher porosity and better antimicrobial properties, but mechanical properties were reduced within an acceptable range. These results indicate that the incorporation of bioactive glass into bone cements can increase the bioactivity of bone cements and can have applications in the medical field.

### 2.2 Antibacterial prosthesis coating

Bacterial infection is a major reason for joint replacement failure (Palierse et al., 2021a). In order to stop bacterial adhesion and counteract joint prosthesis infection, choosing a prosthetic coating with antimicrobial properties can be effective in actively killing bacteria and thus preventing infection. With the development of cell biology and material science, many new antimicrobial prosthetic coatings have been developed and good progress has been made.

#### 2.2.1 Sliver (Ag)

Prior to the discovery of antibiotics, the antibacterial element silver (Ag) was widely employed as a bactericide (Chopra, 2007). The principle of silver sterilization is that Ag cations (Ag^+^) bind to proteins, enzymes, and cell membrane components in bacteria, reacting to destroy them and replace metal ions (such as Zn^2+^ and Ca^2+^), thereby inducing bacterial death (Hetrick and Schoenfisch, 2006). Additionally, silver ions enter the bacterial cell and attach to the sulfhydryl groups of metabolic enzymes in the DNA and electron transport chain, altering bacterial replication and metabolic activities. Bacterial death may result from this (Feng et al., 2000).

With the advancement of technology, silver nanoparticles (AgNPs) are now mostly used instead of traditional metallic silver. Compared with metallic silver or its salts, The benefit of AgNPs is that the antibacterial activity lasts longer and the release of silver ions is gradual and regulated (Nemeno et al., 2014). Currently, there has been some progress in the research and application of coatings for various silver-containing prostheses. Croes et al. (2018) developed a coating formed by combining Chitosan (CS) with AgNPs and showed its antimicrobial effect and sustained drug release properties. There are combinations of silver and calcium phosphate coatings that allow prosthetic coatings to acquire anti-microbial properties, such as Ag-containing hydroxyapatites (HA) nanocrystals that have shown antibacterial activity against *S. aureus* and *E. coli in vitro* (Rameshbabu et al., 2007). Were shown to inhibit Methicillin-resistant *Staphylococcus aureus* (MRSA) colonization and adhesion in a rat *in vivo* research (Shimazaki et al., 2010). One study mixed AgNPs with heparin and chitosan to form an electrolyte coating with antibacterial activity against *E. coli* (Fu et al., 2006). The incorporation of Ag into anti-wear ceramics (e.g., titanium nitride (TiN) (Kelly et al., 2010) and titanium carbon nitride (TiCN) (Sánchez-López et al., 2012), which can reduce the friction of the prosthetic joint coating and reduce debris from joint collisions) can enhance their antimicrobial activity.

Although the silver coating has good sterilization properties, the operation of joint prostheses also requires high loads. Therefore, the mechanical properties of silver-containing coatings on orthopedic implants also need to be taken into account.

#### 2.2.2 Nitric oxide

NO is a gas that has a significant physiological function in organisms. As signal molecules, involved in immune regulation, wound healing, etc (Hoang et al., 2018). It also has some antibacterial activity. It hinders bacterial adherence and has bactericidal effects on both gram-positive and gram-negative bacteria (Charville et al., 2008). It combines with the strong oxidizing agent superoxide (O^2−^) produced by macrophages to create peroxynitrite. This substance can damage cell membranes and also has a toxic effect on bacteria by oxidizing cellular DNA (Hetrick and Schoenfisch, 2006). However, because to the fact that NO is unstable and difficult to control and fix once released (Yang et al., 2018), it has become difficult to control NO to exert its bactericidal effect.

**FIGURE 2:**
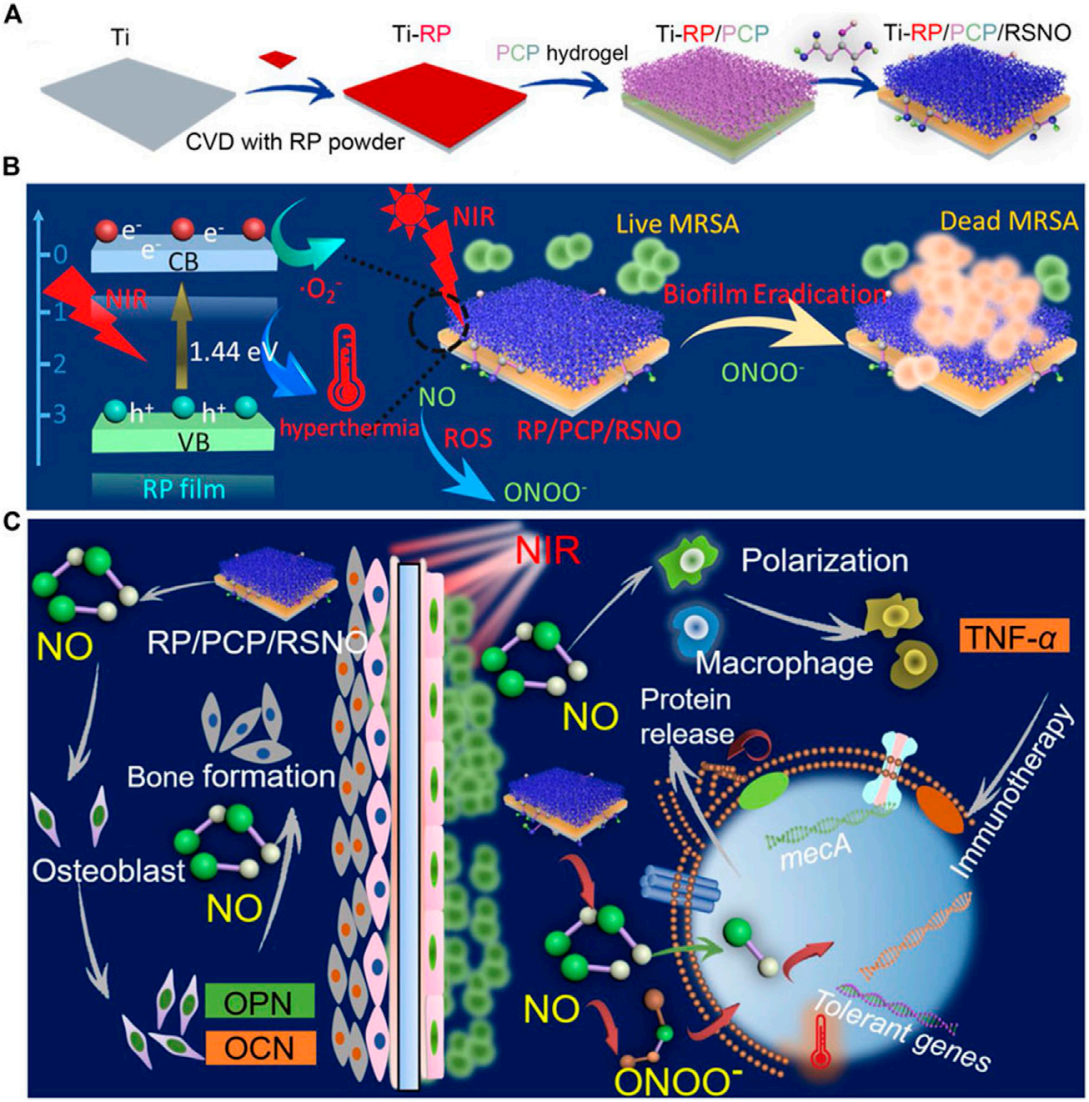
**(A)** Ti-RP/PCP/RSNO hydrogel coating system preparation process schematic diagram. **(B)** A diagram showing how a biofilm can be eliminated using NIR. **(C)** Diagrammatic representation of the processes encouraging bone growth and clearing MRSA biofilms. Ti is for titanium; CVD is for chemical vapor deposition; RP stands for red phosphorus; PCP is for polyvinyl alcohol hydrogel; NIR is for near infrared; MRSA is for methicillin-resistant *Staphylococcus aureus*; and NO is for nitric oxide (Li et al., 2020)., Copyright 2020, American Chemical Society.

A hydrogel system PCP/RSNO made of CS, polydopamine (PDA), and NO-releasing donor modified poly vinyl alcohol (PVA)was created by Li et al. (2020) to address this problem (Figure 2). By coating the hydrogel with a red phosphorous (RP) nanofilm that was deposited on a titanium implant (Ti-RP/PCP/RSNO), the release of NO and O_2_ can be regulated using near-infrared (NIR) light. This resulted in the formation of ONOO^−^. A combination of ONOO^−^, O_2_, and hyperthermia at 808 nm NIR irradiation successfully removed more than 93.1% of an MRSA biofilm, exceeding vancomycin (76.2%). The anti-biofilm efficiency was 91.9% as well. The released NO encouraged osteogenic differentiation and managed inflammatory polarization in addition to serving as an antibacterial against MRSA biofilms by upregulating the expression of phosphatase (ALP), osteocalcin (OCN), osteopontin (OPN) and TNF-α. Bone formation caused by NO released from this coating technology under NIR irradiation was confirmed *in vivo*, with an efficacy rate of 99.2%.

#### 2.2.3 Hydroxyapatite and baicalein

Baicalein is a chemical that is generated from plants and has antibacterial and antioxidant effects (Dinda et al., 2017). Hydroxyapatite as a material with significant biological activity and a molecular structure similar to bone minerals (Yamamuro et al., 1995), has a common clinical application as a coating for prostheses (Kazemi et al., 2020). Palierse et al. (2021b) attempted to investigate the combination of hydroxyapatite nanoparticle coatings with baicalein for improving the properties and antimicrobial activity of prosthetic implants. They used an aqueous solution of calcium, phosphate, sodium, and magnesium salts to create a calcium-deficient biomimetic hydroxyapatite planar coating that was placed on Ti6Al4V alloy and adsorbed with baicalein. The top surface of the hydroxyapatite covering was partially dissolved/remodeled after immersion of the titanium alloy in a baicalein solution. X-ray Photoelectron Spectroscopy and fluorescence analyses were used to assess the presence of adsorbed baicalein on the hydroxyapatite layer despite the inability to quantify it accurately. The findings revealed that *Staphylococcus* epidermidis was significantly inhibited by planar coatings’ antibacterial activity. Nanoparticles treated with baicalein showed strong antioxidant qualities. This result illustrates the ability of hydroxyapatite to adsorb some bioactive molecules, thus enhancing its antibacterial activity.

#### 2.2.4 Gallium-containing active material coating

**FIGURE 3:**
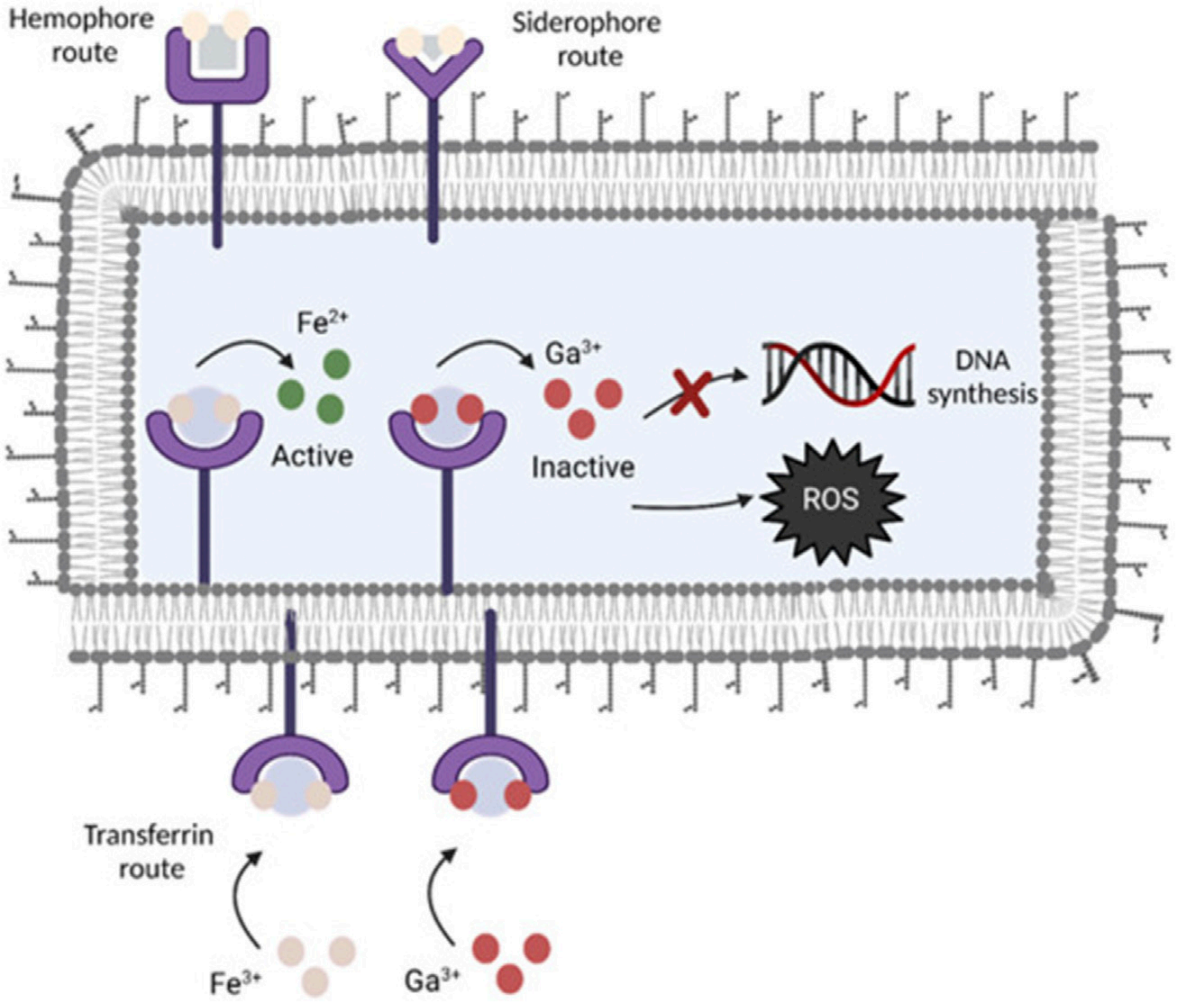
Gallium’s antimicrobial action is shown schematically. By utilizing transferrin, homophora, and siderophore as Fe-uptake pathways, Ga is able to get through the cytoplasmic membrane of bacteria. Because gallium cannot be reduced, it interferes with vital biological processes like respiration, DNA synthesis, and oxidative stress reactions (Kurtuldu et al., 2022). Copyright 2022, Bioactive Materials.

Gallium, as a metal, plays a specific role in the medical field. Gallium ions have some biological activity and have therapeutic effects on some diseases such as hemostasis (Pourshahrestani et al., 2017) and prevention of bacterial infections (Bastos et al., 2019). Gallium is a metallic element with an oxidation state of +3 that belongs to group 13 of the periodic table. Ga^3+^ has some similarities to Fe^3+^ in the body. Cellular respiration, DNA synthesis, and oxygen transport are just a few of the bodily processes that iron is crucial to (Hijazi et al., 2018). When an infection occurs in the body, in response to the immune system, the host decreases the availability of iron so that bacteria lack Fe^3+^ to prevent bacterial proliferation (Andrews et al., 2003), and bacteria also take up large amounts of trivalent iron in response, but the similarity between Fe^3+^ and Ga^3+^ (Xu et al., 2017) makes it difficult for bacteria to distinguish because of the chemical structure. Ga^3+^ and Fe^3+^ are driven into competition for vital bacterial proteins and enzymes as a result. Ga^3+^ cannot be decreased *in vivo* under physiological conditions, in contrast to Fe^3+^, thereby inhibiting the iron-dependent redox pathway within bacteria and acting as an inhibitor of bacterial growth (Figure 3). According to reports, prevalent harmful bacteria like *Pseudomonas aeruginosa* are inhibited by gallium ions (Rimondini et al., 2014; Stuart et al., 2018). The antimicrobial properties of gallium are also used in the coating of many medical implants. Cochise et al. (Cochis et al., 2015) used the anodic spark deposition (ASD) approach to attach gallium to the titanium surface to prevent bacterial development. A simple hydrothermal ion exchange process was used by Yamaguchi et al., (2017) to create a gallium-containing titanium surface coating with a phase of calcium titanate or gallium titanate that contains gallium. Based on the material’s increased bioactivity, the coating’s surface demonstrated an improved antibacterial effect against *Acinetobacter baumannii*. A new technique was created by Dong et al. (2019) to cover the titanium substrates’ surface. The titanium surface was first prepared for TiO_2_ nanotubes by electrochemical anodization. The samples were then submerged in a combination of biodegradable polymer made from poly lactic acid and gallium nitrate. In this method, gallium nitrate is covered by TiO_2_ nanotubes on the surface and offers local transport for Ga^3+^. The endo-energetic layer that was implanted was enough to stop the growth of the *S. aureus* and *E. coli* strains and lessen the inflammatory reaction. Akhtar et al. (2021) devised a method based on the chitosan chelating ability. First, by homogeneously embedding Ga^3+^ ions into chitosan using *in situ* precipitation. Then, the complex was attached to the steel mass by electrophoretic deposition (EPD). Compared to pure chitosan, the Ga^3+^-chitosan complex was 90% more effective in inhibiting *E. coli*.

What’s more, high gallium ion concentrations (50 mg/ml) adversely affect human dermal neonatal fibroblast (HNDF) cells (Best et al., 2020), however, the above study did not show cytotoxicity.

### 2.3 Anti-fouling coating

Biocontamination is caused by the undesirable accumulation of biomolecules, microorganisms, and cells on the wet surface of biomedical implants (Lee et al., 2020).Mitigation of biological For the treatment of periprosthetic infections, the formation of a strongly hydrated layer of tightly adhered hydrophilic macromolecules on the surface has proven to be one of the most effective ways to mitigate biological contamination (Sanchez-Cano and Carril, 2020; Asha et al., 2021). Although polyethylene glycol (PEG) has been widely used for many clinical applications, because it is non-degradable, accumulates in the body and is sensitive to oxidation, this limits the long-term biomedical applications of PEG (Schöttler et al., 2016).So, finding suitable biocompatible materials remains a great challenge.

The development of bioactive antifouling materials is still in its early stages. In the study (Sabaté Del Río et al., 2019; Chang et al., 2022), a novel bioantifouling material was developed based on flexible intrinsically disordered proteins (IDPs) of FUS proteins (fused in sarcoma, a typical RNA-binding protein containing an IDP sequence) containing rich in hydrophilic residues. A chemically defined FUS IDP sequence was labelled using a tetracysteine motif (IDPFUS) and used to covalently immobilise it on different surfaces to form a homogeneous protein entanglement layer, which promoted strong hydration of the surface. *In vitro* and *in vivo* experiments showed that the idpfu-coated surface exhibited good resistance to different cells, platelets, and bacteria. In the study by Lee et al. (2020), DA-HA was synthesised and used as a coating material for fixing to commonly used implantable substrates [(i.e., polyimide (PI), gold (Au), poly (methyl methacrylate) (PMMA), polytetrafluoroethylene (PTFE), and polyurethane (PU)]. Through the self-polymerization of DA-HA at an alkaline pH, highly hydrophilic HA chains were fixed on the substrate surfaces. The surface adherence of cells and non-specific protein adsorption were significantly inhibited on DA-HA-modified substrates. Nearly all macrophage attachment was blocked *in vitro* by DA-HA coating. When compared to the original substrate, non-specific bovine serum albumin (BSA) adsorption on the modified substrate was reduced to 22–46% *in vivo* studies.

These studies show that the use of materials with anti-fouling properties prevents bacterial adhesion to the implant surface and acts as an inhibitor of biofilm growth. They demonstrate the potential for further applications of bioactive materials with anti-fouling capabilities in the biomedical field and provide insight into further exploration of the choice of materials for coating the surfaces of prostheses.

### 2.4 Bioactive glasses

In order to avoid infections in prosthetic joints, substantial research is being conducted on bioactive glasses, a combination of chemicals that serve as a carrier (Rahaman et al., 2014a). They have the capacity to break down in bodily fluids, releasing ions into the environment, while *in vivo* converting to hydroxyapatite (HA), which securely adheres to bones and soft tissues. The process (Jones, 2013) consists of four major phases. 1) the bioactive glass slowly dissolves, allowing soluble ions (including calcium and other ions) to slowly dissolve into the bodily fluid. 2) The development of an amorphous calcium phosphate (ACP) layer on the glass’ surface as a result of the interaction between phosphate ions present in bodily fluid and calcium ions dissolved from the glass. 3) Ongoing ion dissolution in the biogenic glass and delayed ACP layer development. 4) Slow chemical activity gradually converting the ACP layer to HA.

Given the unique properties of bioactive glass as a carrier system, both physically and chemically, it has scalable functional properties in the treatment of joint infections. For enhancing the antimicrobial activity of bioactive glass, three methods are now being investigated**.** The first method focuses on the use of specially designed bioactive glass that, through the glass’s disintegration, modifies the local physiological environment and has a bactericidal impact. The second technique involves adding trace elements to the bioactive glass that have some antibacterial action and may be released through the deterioration of the glass at a pace suited to clinical requirements. Examples of such trace elements include silver. The third technique involves mixing certain antibiotics with bioactive glass, which can operate as a carrier system to disperse the antibiotics across a wide region (Rahaman et al., 2011).

#### 2.4.1 Degradation of specially formulated bioactive glasses leading to changes in the local physiological environment produced (S53P4)

A silicate bioactive glass named 45S5 and bioactive glasses based on 45S5 components (e.g., S53P4 and 13–93) have been most extensively studied for medical applications in bioglass (Rahaman et al., 2014a). S53P4 can achieve antimicrobial effects through changes in the local physiological environment and the release of related combinations. The local pH of simulated bodily fluid may be raised from 7.4 to 11.7 in just 8 h thanks to the dissolution of alkali metal and alkaline earth ions in S53P4 glass, and it can stay there for at least 48 h (Zhang et al., 2006). In a study by Cunha et al. (2018), the antibacterial activity of Bioactive Glass (BAG)-S53P4 was evaluated by time-kill curves against strains associated with joint infections and vancomycin- and gentamicin-resistant strains, and compared with antibiotic-loaded PMMA. The antibacterial capabilities of BAG were found to be comparable to those of the antibiotic-loaded PMMA and to exhibit good *in vitro* antibacterial activity, thus demonstrating the usefulness of BAG-S53P4 as a bioactive bone substitute for antibacterial purposes.

#### 2.4.2 Bioactive glass doped with antimicrobial agents

Silver (Ag) has a long history in the antimicrobial field as an antimicrobial material, used as a frequent biocide before antibiotics were discovered (Chopra, 2007). When creating bioactive glasses, silver may be included into the structure of the glass, and when the glass breaks down, Ag ions are released at a controlled rate. Bioactive glasses with silver doping have shown great success in preventing infections around prosthetics. By using the traditional melting and casting procedure, Luo et al. (2010) created borate bioactive glasses doped with 0.75–2.0 wt% Ag_2_O and assessed the impact of the Ag_2_O concentration on the kinetics of Ag release and biocompatibility. It was discovered that the beginning concentration of Ag_2_O in the glass had an effect on the rate of Ag release. The biocompatibility of bioactive glasses containing 1 wt% Ag_2_O was demonstrated by the cultivation of mouse osteoblasts and fibroblasts in medium containing glass dissolution products. In a study by Prabhu et al. (2013) it was found that the bioactive material glass doped with Ag_2_O nanoparticles exhibited outstanding antibacterial action against *Escherichia coli* and *Staphylococcus aureus*. Besides melting and casting methods, Ferraris et al. (2017) used the *in situ* reduction of silver nanoparticles (Ag-NPs) with antibacterial properties on bioactive glass. They used the reduction of silver ions on the surface of the glass to metallic silver nanoparticles and evaluated the ability of the bioactive glass against S. aureus. It was found that the bioactive glass containing Ag-NPs had a 20% increase in the antibacterial ability against S. aureus. What’s more, it was shown that while physiologically active, Ag_2_O was somewhat hazardous to human cells at a concentration of 100 μg ml ^(−1)^ (Prabhu et al., 2013). In another study by Ryan et al. (2022), mice were given daily oral doses of AgNPs^+^ (20.5 mg kg^−1^) and Ag^+^ (20.5 mg kg^−1^). 18 days later mice showed hepatomegaly and splenomegaly.

Copper (Cu) has an important role as a trace element in the human body, and nowadays it also has been shown to have an effect on angiogenesis and to be a strong antimicrobial agent (Bharathi Devi et al., 2016). Rivera et al. (2022). Developed an innovative surface coating by electrophoretic deposition (EPD) consisting of a copper-containing bioactive glass and some bioactive materials. The coating produced by EPD was found to have significantly higher (*p* < 0.05) properties against combined bacteria like *Staphylococcus aureus*, *Staphylococcus* epidermidis, and *Escherichia coli* antibacterial compared to the copper-free coating after images under field emission scanning electron microscopy (FESEM) were examined. Additionally, it was discovered that when cells and bacteria were cultivated together on this coated surface, cell viability was sustained. Finally, *in vivo* research showed that *S. aureus* biofilm growth was inhibited, and the development of neovascularization (<50 μm diameter) was noted. Thus, it was proven that elemental copper may both encourage angiogenesis and prevent bacterial infection. In another study by Miola and Verné (2016), they used copper doping of bioactive glass powders in aqueous solution by ion exchange techniques. In the ion exchange process, they first used different copper salts (nitrate, chloride, acetate and sulphate) at the same concentration and found in the structural analysis that ion exchange with copper nitrate and copper chloride produced a crystalline phase in the bioactive glass and that the bioactive glass ion exchanged with copper acetate contained more copper than that exchanged with copper sulphate. The ion-exchange technique was then used with different concentrations of copper acetate to test the antibacterial activity of bioactive glass powders ion-exchanged in copper acetate solution and it was found that the glass powders ion-exchanged in 0.05 M copper acetate solution had a good antibacterial effect against *S. aureus*, meanwhile no crystalline phase was generated. The biological activity of the glass was not affected and the antibacterial effect of copper in bioactive glasses was thus confirmed.

#### 2.4.3 Biologically active glasses loaded with antibiotics

Because they are often dense (have few or no pores) and cannot be used as a delivery system for antibiotics, bioactive glasses made using normal fusion casting techniques are not suited. Use of a composite delivery system, which consists of a biodegradable biosubstrate containing antibiotics and bioactive glass particles scattered throughout it, is one remedy. While the system is given biological activity by the bioactive glass, the antibiotic is released as the matrix breaks down. Current research on degradable biomatrices composed of phosphate cements (Xie et al., 2009) or organic phases (polymers or hydrogels) (Rahaman et al., 2014b) has progressed.

In one experiment, phosphate cements containing vancomycin and composites made of borate bioactive glass particles were submerged in simulated body fluid (SBF), and vancomycin was released rapidly in the first 8–10 days (Xie et al., 2009) and in about 85% of the initial total amount in the first 10 days. Subsequently, the release slows down significantly and stops after 14 days, when the cumulative release of vancomycin is 90% of the initial amount.

In another study (Rahaman et al., 2014b), teicoplanin-containing glass particles attached to chitosan in a composite were submerged in phosphate-buffered saline (PBS). The antibiotic release was 45–50% of the initial total after 1 day of immersion and 70–75% of the antibiotic release after 7 days. Subsequently, the rate of antibiotic release decreases significantly and stops after about 21 days, at which point the cumulative release of teicoplanin is 80–85% of the initial amount.

### 2.5 Hydrogels

A hydrogel is a polymer network consisting of hydrophilic polymers with high water content. Hydrogels are naturally better at adhesion, *in situ* polymerization, and prolonged drug release when it comes to drug delivery (De Witte et al., 2018). Currently, *in vitro*, in preclinical models, and in human trials, hydrogels are being studied as antibiotic transporters. By adding several antibiotics *in vitro*, hydrogels’ capacity to guard against infection in orthopedic implants was demonstrated (Drago et al., 2014).

The variety of antibiotics has increased as a result of the use of hydrogels as drug carriers. Despite the fact that the medication lowers the lower critical dissolving temperature (LCST) of thermally sensitive hydrogels (such as HA-pNipam hydrogels) due to the Hofmeister effect (Ter Boo et al., 2016), hydrogels loaded with antibiotics are still injectable and gellable, and their applicability and payload are not affected. Therefore, there is a study of incorporating broad-spectrum antimicrobial agents into hydrogels (Boot et al., 2021) with good efficacy in animal models.

Gentamicin and vancomycin, two of the most effective antibiotics for treating joint infections, can be given in a variety of ways, and the release of antibiotics can be tailored at various rates depending on the carrier system (burst, sustained, burst and sustained). Vancomycin’s structure may be altered by direct release because of interactions between its positive charge and the negatively charged alginate (ALG) chain (Jung et al., 2019). In research by Li et al. (2017), vancomycin was contained in a hydrogel membrane made of poly ethylene glycol (PEG), which was covalently attached to titanium implants. Vancomycin was found to consistently release *in vivo* for more than 4 weeks, without any initial abrupt release, and to have good anti-microbial activity against *S. aureus*. In another study, according to Foster et al. (2021) reported that the HA-pNipam hydrogel may release the antibiotic combination (gentamicin and vancomycin) for more than 10 days, and the ultimate local concentration might be greater than the MIC. These studies suggest that hydrogels as carriers are capable of sustained release of antibiotics to achieve effective bacterial inhibition.

### 2.6 Protamine

Protamine is a highly polycationic AMP that is generated from fish sperm and is an arginine-rich protein that has antibacterial properties (Honda et al., 2020). The principle is that protamine is positively charged and the bacterial membrane is negatively charged, and the electrostatic interaction between the two can drive the expression of protamine bactericidal activity. Protamine acts as an alkaline molecule that forms complexes with acidic molecules including DNA, heparin, and flagellin by interacting with them *via* ions (Ishihara et al., 2015). Nanoparticle produced by electrostatic action can act as fixators or attractants for various cytokines, inhibiting or killing many microorganisms, including bacteria and fungi. These nanoparticles/particles have been utilized in biomaterials such as transporters for cells, proteins, growth factors, and others (Nemeno et al., 2014). Honda et al. (2020) develop a new bone tissue engineering material based on hydroxyapatite containing protamine (protamine/HAp). It was protamine/HAp powders that have capablity of releasing protamine and inhibiting bacterial growth *in vitro*. So it can be seen that in the biomedical field, protamine is becoming more and more important in tissue engineering and regenerative medicine and can be used as an alternative to antibiotics.

In summary, there are promising applications for bioactive materials in the prevention of pji, and the applications, methods and advantages and disadvantages of the previously listed materials are listed in Table 1.

**TABLE 1:** Summary of advantages and disadvantages of different bioactive materials in prevention of PJI.

Application	Bioactive materials	Method	Advantages	Disadvantages	Refs
Bone cement	Quaternary ammonium and alkoxysilane	Add γ-methacryloxypropyltrimetoxysilane to provide the biological activity of PMMA cement and use 2-(tert-butylamino) ethyl methacrylate (TBAEMA) to increase antimicrobial performance	Exhibit antimicrobial properties against both gram-positive and gram-negative bacteria	As the TBAEMA content increased, the compressive strength decreased	Wang et al. (2021)
	Chitosan and graphene oxide	Add nanosheets composed of GO and CS	The ABC nanocomposites made with 15% CS and 0.3% GO (CS + GO) have a synergistic impact on physical, mechanical, thermal, and antibacterial characteristics, all ABCs were free of cytotoxicity and supported good cell viability of human osteoblasts (hob)	The addition of CS decreased the compression properties of ABCs	Valencia Zapata et al. (2019)
Bioactive glass	Adding bioactive glass to bone cement	Bioactive glasses incorporated with smaller particles (40 μm) exhibited higher porosity and better antimicrobial properties	Mechanical properties were reduced within an acceptable range		Nemeno et al. (2014)
Antibacterial prosthesis coating	Sliver (Ag)	A coating formed by combining Chitosan (CS) with agnps	Chitosan-based Ag coating completely eliminates *Staphylococcus aureus* by up to 4 orders of magnitude	Antibacterial Ag concentrations were cytotoxic for neutrophils, and that non-toxic Ag concentrations diminished their phagocytic activity	Wekwejt et al. (2021)
Combinations of silver and calcium phosphate coatings	The coating showed antibacterial activity against *S. Aureus* and *E. Coli in vitro*	-			Rameshbabu et al. (2007)
Place Ag-containing HA nanocrystals on the surface of titanium implants	The mean number of surviving MRSA on HA coatings was (1.5±0.5) x 10 (5), significantly higher than (1.1±0.4) x 10 (4) on Ag-HA coatings (*p* < 0.001)	Serum Ag ion concentrations rise to 36 times the expected clinical standard for a short period of time and then gradually decline to normal levels			Shimazaki et al. (2010)
Mix agnps with heparin and chitosan to form an electrolyte coating	The chitosan/heparin multilayer coating were possessed of bactericidal effect on *E. Coli* and did not show any cytotoxicity	-			Fu et al. (2006)
Nitric oxide (NO)	Use CS, PDA and NO release donor modified PVA to make a Hydrogel system PCP/RSNO	The Ti-RP/PCP/RSNO coating can remove more than 93.1% of an MRSA biofilm.The anti-biofilm efficiency was 91.9% as well. The released NO encouraged osteogenic differentiation and managed inflammatory polarization in addition to serving as an antibacterial against MRSA biofilms, and the coating was innocuous and efficient *in vivo*	-		Li et al. (2020)
Hydroxyapatite and baicalein	Place hydroxyapatite planar coating on Ti6Al4V alloy and adsorbed with baicalein	High antioxidant activity and significant antibacterial activity	Low antibacterial activity		Palierse et al. (2021b)
Gallium	Use the anodic spark deposition (ASD) approach to attach gallium to the titanium surface	Gallium-coated samples showed higher inhibition values compared to controls (gacis: 40% and gaoss: 48%), both without any direct or indirect cytotoxic effects	-		Cochis et al. (2015)
	Create a gallium-containing titanium surface coating with a phase of calcium titanate or gallium titanate that contains gallium	The coating’s surface demonstrated an improved antibacterial effect against *Acinetobacter baumannii*	-		Yamaguchi et al. (2017)
	The titanium surface was first prepared for Tio2 nanotubes by electrochemical anodization. The samples were submerged in a combination of biodegradable polymer made from poly lactic acid and gallium nitrate	Stop the growth of the *S. Aureus* and *E. Coli* strains and lessen the inflammatory reaction	-		Dong et al. (2019)
	Synthesize Gallium (III) -chitosan (Ga (III)-CS) complexes with six different concentrations of gallium by *in situ* precipitation	The Ga^3+^-chitosan complex was 90% more effective in inhibiting *E. Coli* than pure chitosan	Higher concentrations of Ga (III), i.e. 1:2 and 1:1 Ga (III)-CS complexes, resulted in reduced viability of human osteosarcoma cell line and mouse embryonic fibroblasts, but were still above 80%		Akhtar et al. (2021)
Anti-fouling coating	Dopamine-hyaluronic acid conjugates	The highly hydrophilic HA chains are immobilised on the surface of the material by the self-polymerisation of DA-HA at alkaline ph	The surface adherence of cells and non-specific protein adsorption were significantly inhibited on DA-HA-modified substrates. No significant inflammatory reaction to the implant	-	Lee et al. (2020)
	Intrinsically Disordered Protein Condensate	A novel bioantifouling material was developed based on flexible intrinsically disordered proteins (idps) of FUS proteins (fused in sarcoma, a typical RNA-binding protein containing an IDP sequence) containing rich in hydrophilic residues	Idpfu-coated surface exhibited good resistance to different cells, platelets, and bacteria	Show low cytotoxicity	Chang et al. (2022)
Bioactive Glasses	Bioactive Glass (BAG)-S53P4	Specially formulated bioactive glasses	The antibacterial capabilities of BAG were found to be comparable to those of the antibiotic-loaded PMMA and to exhibit good *in vitro* antibacterial activity	-	Cunha et al. (2018)
	Sliver (Ag)	Doping of silver in bioactive glass by melting and casting	Bioactive glasses containing 1 wt% Ag_2_O has good biocompatibility	Ag_2_O was somewhat hazardous to human cells at a concentration of 100 μg ml^−1^.Mice would show hepatomegaly and splenomegaly given daily oral doses of agnps+ (20.5 mg kg^−1^) and Ag^+^ (20.5 mg kg^−1^)	Luo et al. (2010)
			The bioactive material glass doped with Ag_2_O nanoparticles exhibited outstanding antibacterial action against *Escherichia coli* and *Staphylococcus aureus*		Prabhu et al. (2013)
		Using the *in situ* reduction of silver nanoparticles (Ag-NPs) with antibacterial properties on bioactive glass	The bioactive glass containing Ag-NPs had a 20% increase in the antibacterial ability against *S. aureus*	-	Ferraris et al. (2017)
	Cooper (Cu)	Antimicrobial agents	Copper may both encourage angiogenesis and prevent bacterial infection	Large doses of copper are toxic and cause disease (cytotoxicity was not demonstrated in this study)	Rivera et al. (2021)
		Using copper doping of bioactive glass powders in aqueous solution by ion exchange techniques	The glass powders ion-exchanged in 0.05 M copper acetate solution had a good antibacterial effect against *S. aureus*	Excess copper ions are cytotoxic, but this study did not show cytotoxic effects on osteoblast-like cells	Miola and Verné, (2016)
	Vancomycin	Bioactive Glasses loaded with antibiotics	Antibiotics are released rapidly and in large quantities in the short term and slowly in the later stages to meet antibacterial requirements	Temporarily high concentrations of topical antibiotics may lead to toxicity	Xie et al. (2009)
	Teicoplanin				Rahaman et al. (2014b)
Hydrogels	Vancomycin	Vancomycin was contained in a hydrogel membrane made of poly ethylene glycol (PEG), which was covalently attached to titanium implants	Consistently release *in vivo* for more than 4 weeks, without any initial abrupt release, and to have good anti-microbial activity against *S. Aureus*	-	Li et al. (2017)
	Ntibiotic combination (gentamicin and vancomycin	Use HA-pnipam hydrogels as drug carriers	Release the antibiotic combination for more than 10 days, and the ultimate local concentration might be greater than the MIC	Temporarily high concentrations of topical antibiotics may lead to toxicity	Foster et al. (2021)
Powder	Protamine	Hydroxyapatite containing protamine (protamine/hap)	Have capablity of releasing protamine and inhibiting bacterial growth *in vitro*	Viability of cell cultures exposed to high doses of protamine/hap (over 1000 μg ml^−1^) induced human osteoblastic cells (MG-63) death	Honda et al. (2020)

## 3 Bioactive materials in detection of PJI

The diagnostic criteria for PJI are still debated and are commonly used for hematology, joint fluid examination, imaging, histopathology, and culture and molecular diagnosis of pathogenic microorganisms (Bistolfi et al., 2019). Serum ESR (erythrocyte sedimentation rate) and CRP (C-reactive protein) are reliable predictors among blood tests; however, they are less specific for infections than serum ESR and CRP. Joint fluid examination, histopathological examination and culture and molecular diagnosis of pathogenic microorganisms can cause new contamination because they are invasive tests. Imaging examinations are difficult to detect early PJI conditions. So new strategies have to be found regarding the methods of diagnosing PJI.

### 3.1 Hydrogel pH sensors

A water-soluble polymer substance called hydrogel has the capacity to reversibly alter volume in response to various environmental conditions (such as changes in pH, temperature, electric field and light) (Caló and Khutoryanskiy, 2015). Acrylic polymers, with their excellent biocompatibility and long lifetime, have a variety of uses in biosensors and medication administration (Caló and Khutoryanskiy, 2015).

**FIGURE 4:**
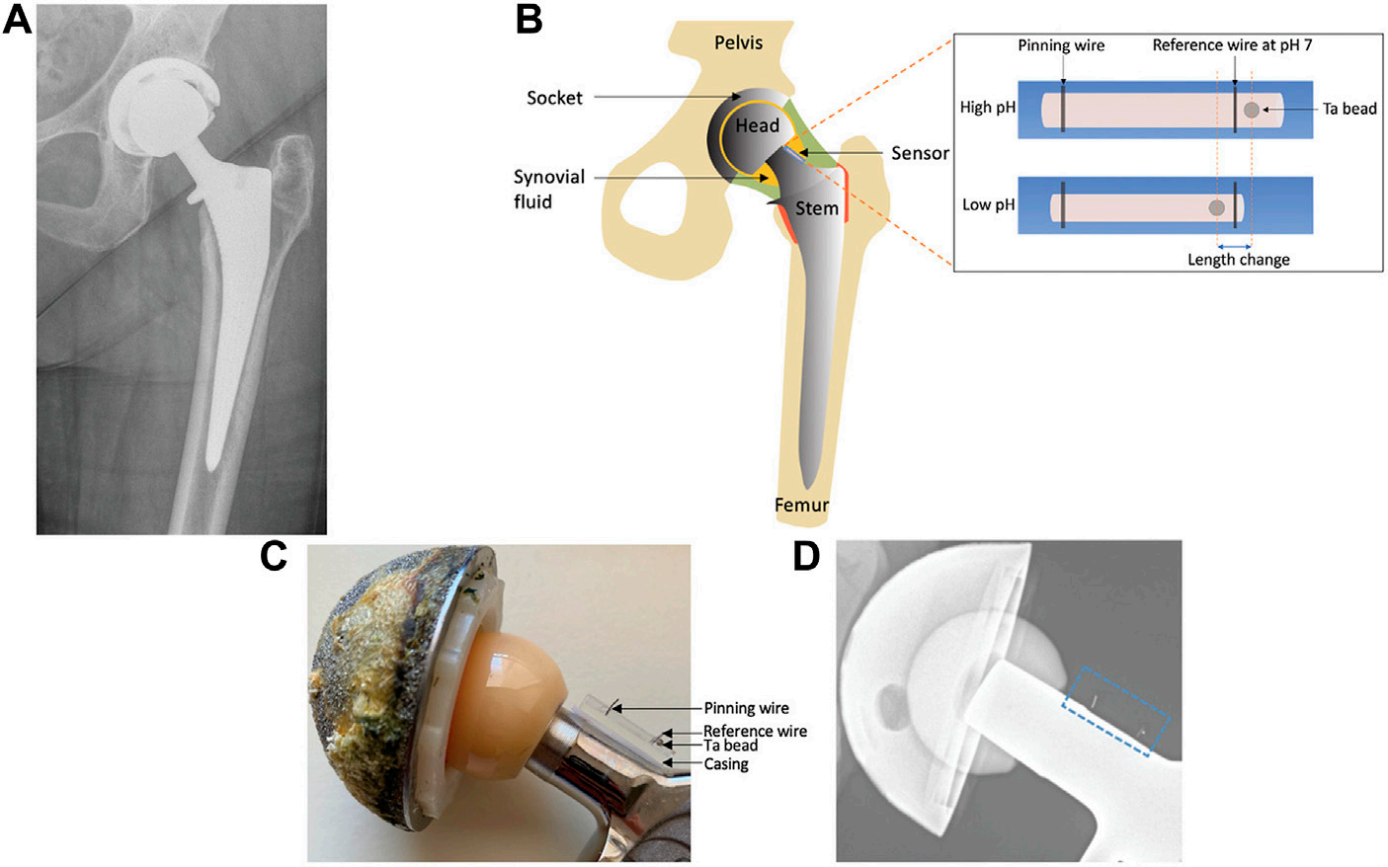
**(A)** X-ray pictures of people who have artificial hip joints. **(B)** A prosthetic hip joint with a synovial pH sensor is shown schematically. **(C)** Image of a hip prosthesis with an associated pH sensor. **(D)** Photograph of a hip prosthesis with a pH sensor attached (Wijayaratna et al., 2021). Copyright 2021, Advanced Functional Materials.

According to several researches, when compared to normal synovial joint fluid, the pH of synovial fluid drops from 7.5 under sterile settings to 6.7–7.0 during infection (Ward and Steigbigel, 1978; Lenski and Scherer, 2015). In addition, pH is associated with leukocyte counts (Ward and Steigbigel, 1978; Park and Kim, 2013) and lactate (Brook et al., 1978) are closely related. Both of them can be used to detect artificial joint infections. Based on these principles, Professor Jeffrey N. Anker’s team designed and developed an implantable sensor that can measure the pH of synovial fluid in the joint (Wijayaratna et al., 2021), enabling early identification and surveillance of hip infections with standard flat films. The pH-responsive polyacrylic acid hydrogel used in the sensor will swell at high pH levels and contract at low pH levels. Plain radiography may assess the location of the radio-dense tantalum beads in the hydrogel relative to the radio-dense scale adjacent to the hydrogel and report the pH in it because high-density tantalum beads and tungsten wires are inserted in the ends of the hydrogel (Figure 4). Prior to surgical implantation, the sensor is fastened to the prosthesis. It uses simple X-rays to offer a painless, non-invasive, quick, and affordable assessment.

Anker’s team reports the first implantable sensor to measure the pH of synovial fluid using a flat sheet. With a pH precision of 0.08 pH units, the sensor responds linearly and is reversible over a period of 30 min in solutions of bovine synovial fluid with a pH between 6.5 and 7.5. The technique employs X-rays, which are already a part of the accepted standard of care in the post-operative context, and is quick, non-invasive, and accurate. As a result, the created sensor has the potential to be employed as a chemical sensor that can detect periprosthetic joint infections using X-ray imaging (Wijayaratna et al., 2021).

### 3.2 Fluorescence labelled polymeric nanoparticles

One of the most often used compounds for *in vivo* imaging is fluorescent dye, which has been applied extensively in both scientific and therapeutic settings (Dunst and Tomancak, 2019). Fluorescent dyes have the ability to exhibit excitation properties at certain wavelengths and emit light at different wavelengths (Guo et al., 2014), which can serve as both qualitative or quantitative diagnostic probes and can be of great value for the diagnosis of diseased cells and tissues *in vivo* based on *in vivo* imaging (Smith and Gambhir, 2017), with the advantages of rapid diagnosis, low volume and high sensitivity, no radiation and low toxicity (Fang et al., 2017). Conjugated polymers (CPs) are biomolecules with photoluminescence, light absorption, and electrical conductivity (Jin et al., 2015). According to its properties, CPs capable of exhibiting fluorescent properties have recently been widely used in the focus of bioimaging, especially non-invasive *in vivo* imaging (Feng et al., 2010).

Based on the characteristics of fluorescent dyes and CPs, Norouz Dizaji et al. (2020) reported the following study: They used maleimide functionalized 1,2-distearoyl-sn-glycero-3-phosphoethanolamine-N-(maleimide (polyethylene glycol)-2000) as the main carrier matrix to prepare nanoparticles (NPs) and selected poly (9,9′-bis(6″-N,N,N-trimethylammonium) hexyl) fluorene-co-alt-4,7-(2,1,3-benzothiadiazole) dibromide), a CPs exhibiting fluorescent properties, was chosen as the probe. The CPs were encapsulated in these NPs using ultrasound, and synthetic thiolated vancomycin was attached to the NPs. They then conducted *in vivo* live tracing studies by a ‘non-invasive live animal fluorescence imaging technique’ using Maestro EX Fluorescence Imaging System (CRi, Inc.,Waltham, MA, United States). In their studies, these unique nanoparticles contain optical and specific targeting agents that effectively test MRSA bacterial infections through non-invasive *in vivo* imaging, meaning that specific targeting agents can be applied to other targets by altering them. The findings demonstrate that polymeric nanoparticles labeled with fluorescence may be used to successfully detect bacterial infections *in vivo*.

In the detection of PJI, although the current clinical approach to PJI is still predominantly invasive, hydrogel pH sensors and fluorescence labelled polymeric nanoparticles offer new ways of thinking about non-invasive PJI detection.

## 4 Bioactive materials in treatment of PJI

PJIs are often treated by removal of the infected prosthesis, removal of bone pieces that are necrotic, topical and/or systemic antibiotics, and joint restoration with a new prosthesis as soon as the infection is under control (Costa-Pinto et al., 2021). Although patients with PJI undergo rigorous treatment, at much expense, it is often difficult to achieve treatment outcomes in uninfected TJA patients. The resistance of bacterial biofilms to mechanical debridement and medicines is a significant factor in this (Levack et al., 2018). Due to their biocompatibility, biodegradability, and minimal immunogenicity, bioactive materials have significant research implications in the treatment of PJI (Costa-Pinto et al., 2021).

### 4.1 Hydrogel materials loaded with antimicrobial agents

Hydrogels have the advantage of adhesion and sustained drug release in terms of drug transport (De Witte et al., 2018), yet in terms of substance removal, hydrogels have degradable properties (such as collagen and hyaluronic acid (Kasugai et al., 2000)) and will be degraded by the host to be replaced by the repair tissue, allowing removal of hydrogels without additional surgery (Johnson et al., 2018). These characteristics allow hydrogels to be used for antimicrobial delivery under different complex anatomical conditions (Slaughter et al., 2009), allowing antimicrobial drugs (mainly antibiotics) to be used not only for the prevention of PJI, but also for its treatment. *In vivo* studies of different antimicrobial agents for the treatment of PJI on hydrogel-based drug delivery systems have yielded promising results.

#### 4.1.1 Antibiotics

Hydrogels are excellent carriers of antibiotics, not only for PJI prophylaxis, but also for the treatment of PJI according to its infection. As mentioned earlier, hydrogels loaded with antibiotics have injectable and gelling properties and do not affect their applicability and payload. In addition to this, studies have also demonstrated the efficacy of incorporating broad-spectrum antimicrobial agents into hydrogels in animal models (Foster et al., 2021) to inhibit bacterial growth.

However, the extensive use of antibiotics has caused a large number of bacteria to become resistant to their usage (Roca et al., 2015), which implies a greater use of antimicrobial agents instead of antibiotics, which has sparked the development of therapeutic modalities outside from conventional antibiotic treatment. Currently, for the treatment of PJI, several biologic antimicrobial drugs, including phage and lysostaphin, are used in variable degrees in topical administration systems with hydrogels.

#### 4.1.2 Lysostaphin

Lysostaphin is a metallo-endopeptidase produced by S. simulans. This bacteriolytic enzyme is characterized by a highly selective anti-staphylococcal action (Kumar, 2008). The significant anti-staphylococcal activity of this bacteriolytic enzyme also showed antibacterial efficacy against strains that were resistant to medication (including MRSA, vancomycin-resistant *Staphylococcus aureus*, and *Staphylococcus* epidermidis) (Mohamed et al., 2014). Lysozyme is able to effectively kill bacteria at lower concentrations (Wu et al., 2003) and the antimicrobial activity of lysozyme is independent of the metabolic state of the bacteria, which allows it to also have an antimicrobial effect against bacteria in biofilms (Patron et al., 1999). Additionally, human cell osteogenic differentiation is unaffected by lysostaphin (Johnson et al., 2018). Due to these qualities, lysostaphin is a potential therapy for PJI brought on by staphylococcal infections.

Johnson et al. (2018) created a low-toxicity, injectable four-arm PEG macromers functionalized with terminal maleimide groups (PEG-4MAL) hydrogel by fusing the traits and qualities of lysostaphin and hydrogel. They found that the addition of lysozyme had no effect on the hydrogel’s elasticity or adhesive qualities, and that the hydrogel’s reticular structure could be altered to regulate the release of lysozyme. They conducted studies in a mouse model and found that PEG-4MAL hydrogels containing lysostaphin were able to clear infection and support fracture healing or defect repair. Thus, it was demonstrated that lysostaphin can be applied to treat PJI caused by staphylococcal infections.

#### 4.1.3 Phages

Phages, as an invasive bacterial virus, are highly specific and inhibit most known bacteria (Petrovic Fabijan et al., 2020). Thus, it has a sustained antibacterial effect and prevents and controls bacterial biofilm formation (Geredew Kifelew et al., 2019). Wroe et al. (2020) designed an injectable hydrogel based on PEG-4MAL by wrapping P. aeruginosa phage in hydrogel and injecting it into the joint infection site. The phage was able to release in a controlled manner and retain its bacteriolytic activity. Compared to the phage-free hydrogel group, the live P. aeruginosa count in the phage-containing hydrogel group was reduced by 4.7-fold 7 days after implantation at the location of the infected deficiency in mice. In a clinical trial by Petrovic Fabijan et al. (2020), a phage-assisted method for the treatment of serious *S. aureus* infections was reported and evaluated for safety and tolerability. The results did not report any adverse reactions and found that no phage resistance developed *in vivo*. These results demonstrate that the delivery of phages using hydrogels as carriers can provide a new therapeutic direction for PJI.

### 4.2 Targeting nanoparticle-antibiotics delivery vehicle

The use of traditional antibiotic therapies for PJI still has problems that cannot be solved; they do not effectively target the site of bone infection, leading to unsatisfactory treatment outcomes (Zhang et al., 2022). Therefore, bone-targeted and bacterial-targeted therapies have been extensively studied in bone infections.

Peptide D_6_ is an effective carrier molecule for bone-targeted drugs in which aspartic acid has a high affinity for bone tissue (Kasugai et al., 2000). The peptide UBI_29-41_, which has six positively charged residues and is the most widely used bacterial targeting agent, has a strong affinity for *S. aureus* due to its greater negative charge (Chen et al., 2015). It has been demonstrated that UBI_29-41_ can discriminate between aseptic loosening and periprosthetic infection (Beiki et al., 2013). As a result, it is anticipated that UBI29-41 peptide will be employed for clinical localization of infected lesions. In order to target *S. aureus* and regulate the release of vancomycin, Hussain (Hussain et al., 2018) et al. discovered that silica nanoparticles modified with cyclic 9-amino acid peptides could well be utilized. This improved antibacterial activity.

Nie et al. (2022) used a dual bone and bacterial targeting strategy to create a new mesoporous silica nanoparticle (MSN) that targets the location of the bone infection based on the aforementioned concepts. The vancomycin-loaded particles increased the effectiveness of treating orthopedic implant-associated infections by precisely delivering the antibiotic to the location of bone infection. The findings of *in vivo* and *in vitro* tests revealed that the MSNs with dual targeting for bacteria and bone have outstanding antibacterial capabilities *in vitro*. Methicillin-resistant *Staphylococcus aureus* infection caused by an orthopedic implant in a rat model was greatly suppressed by the antibacterial treatment. As a result, the molecularly modified nanoparticles with dual-targeting for bacteria and bone can target the bone location of bacterial infection and allow for the early treatment of PJI.

In the treatment of PJI, the incorporation of broad-spectrum antibiotics and the use of antimicrobial agents in hydrogels are directions for research other than surgery, and new efficacious drugs are yet to be discovered and studied.

## 5 Summary and outlook

This review provides an overview of the various applications of bioactive materials for PJI and thus provides insight into the development of new countermeasures for PJI, one of the most severe post-orthopedic surgical consequences and a significant contributor to surgical failure, leading to difficult revision surgeries and, in extreme cases, amputation and even death. Only a few microbes are needed for PJI to infect the implant, which causes a microbial biofilm to develop on the surface. Traditional treatment methods are complicated and ineffective. Bioactive materials, as potentially valuable biomaterials, are excellent in dealing with PJI because of their unique bioactivity.

In comparison with current clinical therapies, bioactive materials have shown unique advantages in prevention, detection and treatment. There are modifications of existing ones, such as bone cements, bio-coatings, and filler materials, as well as new hydrogel materials, antimicrobial agents, and various antibiotic-loaded nanoparticles, which, in several *in vivo* and *in vitro* investigations, have produced encouraging outcomes. And in the post-antibiotic era, some non-antibiotic antimicrobial therapies are of particular importance. Some bioactive materials (e.g., Silver, Copper, Nitric oxide, Gallium, Protamine, Lysostaphin, etc.) have potential scope for the prevention and treatment of PJI.

Overall, compared to current therapeutic methods to PJI, the accessible bioactive materials have shown greater capacity to prevent, diagnose, and treat PJI. There are still significant difficulties with clinical applications, though. First, the use of most bioactive materials still requires loading with antibiotics, which can lead to resistance in some bacteria. Second, the proper functioning of joint prostheses requires high loading intensities, and the mechanical properties of some bioactive materials need to be considered when applied to joint prostheses. Third, Staphylococcus epidermidis, *Staphylococcus aureus*, and *Pseudomonas aeruginosa* are the most prevalent pathogenic microorganisms for PJI. The antibiotics and antimicrobial agents used in most materials only target common strains of bacteria, and there has been less research on some of the less common pathogenic bacteria. Fourth, to date, only a small number of bioactive materials have been successfully used in clinical settings. More research is needed to develop bioactive materials that can be applied to PJI.

Due to the complicated milieu of PJI, removal of biofilm from the infection site continues to be a significant issue. A better cognition of the mechanisms of PJI, using various properties of bioactive materials, together with the antibacterial effect of antibiotics or antimicrobials, will help to develop and formulate better therapeutic strategies.

## References

[B1] Akhtar M. A. Hadzhieva Z. Ilyas K. Ali M. S. Peukert W. Boccaccini A. R. (2021). Facile synthesis of gallium (III)-Chitosan complexes as antibacterial biomaterial. Pharmaceutics 13 (10), 1702. 10.3390/pharmaceutics13101702 34683993 PMC8541496

[B2] Andrews S. C. Robinson A. K. Rodríguez-Quiñones F. (2003). Bacterial iron homeostasis. FEMS Microbiol. Rev. 27 (2-3), 215–237. 10.1016/s0168-6445(03)00055-x 12829269

[B3] Arciola C. R. Campoccia D. Ehrlich G. D. Montanaro L. (2015). Biofilm-based implant infections in orthopaedics. Adv. Exp. Med. Biol. 830, 29–46. 10.1007/978-3-319-11038-7_2 25366219

[B4] Asha A. B. Chen Y. Narain R. (2021). Bioinspired dopamine and zwitterionic polymers for non-fouling surface engineering. Chem. Soc. Rev. 50 (20), 11668–11683. 10.1039/d1cs00658d 34477190

[B5] Bastos R. W. Rossato L. Valero C. Lagrou K. Colombo A. L. Goldman G. H. (2019). Potential of gallium as an antifungal agent. Front. Cell. Infect. Microbiol. 9, 414. 10.3389/fcimb.2019.00414 31921699 PMC6917619

[B6] Beiki D. Yousefi G. Fallahi B. Tahmasebi M. N. Gholamrezanezhad A. Fard-Esfahani A. (2013). 99m)tc-Ubiquicidin [29-41], a promising radiopharmaceutical to differentiate orthopedic implant infections from sterile inflammation. Iran. J. Pharm. Res. 12 (2), 347–353. 24250609 PMC3813225

[B7] Berend K. R. Lombardi A. V. Jr. Morris M. J. Bergeson A. G. Adams J. B. Sneller M. A. (2013). Two-stage treatment of hip periprosthetic joint infection is associated with a high rate of infection control but high mortality. Clin. Orthop. Relat. Res. 471 (2), 510–518. 10.1007/s11999-012-2595-x 22983683 PMC3549176

[B8] Best M. G. Cunha-Reis C. Ganin A. Y. Sousa A. Johnston J. Oliveira A. L. (2020). Antimicrobial properties of gallium (III)-and iron (III)-Loaded polysaccharides affecting the growth of *Escherichia coli*, *Staphylococcus aureus*, and *Pseudomonas aeruginosa*, *in vitro* . ACS Appl. Bio Mat. 3 (11), 7589–7597. 10.1021/acsabm.0c00811 35019499

[B9] Bharathi Devi S. R. Dhivya M. A. Sulochana K. N. (2016). Copper transporters and chaperones: Their function on angiogenesis and cellular signalling. J. Biosci. 41 (3), 487–496. 10.1007/s12038-016-9629-6 27581939

[B10] Bistolfi A. Ferracini R. Albanese C. Vernè E. Miola M. (2019). PMMA-based bone cements and the problem of joint arthroplasty infections: Status and new perspectives. Mater. (Basel, Switz. 12 (23), 4002. 10.3390/ma12234002 PMC6926619 31810305

[B11] Boot W. Schmid T. D'Este M. Guillaume O. Foster A. Decosterd L. (2021). A hyaluronic acid hydrogel loaded with gentamicin and vancomycin successfully eradicates chronic methicillin-resistant *Staphylococcus aureus* orthopedic infection in a sheep model. Antimicrob. Agents Chemother. 65 (4), 018400-e1920. 10.1128/aac.01840-20 PMC8097416 33526492

[B12] Brook I. Reza M. J. Bricknell K. S. Bluestone R. Finegold S. M. (1978). Synovial fluid lactic acid. A diagnostic aid in septic arthritis. Arthritis Rheum. 21 (7), 774–779. 10.1002/art.1780210706 697948

[B13] Caló E. Khutoryanskiy V. V. (2015). Biomedical applications of hydrogels: A review of patents and commercial products. Eur. Polym. J. 65, 252–267. 10.1016/j.eurpolymj.2014.11.024

[B14] Chang R. Chen J. L. Zhang G. Y. Li Y. Duan H. Z. Luo S. Z. (2022). Intrinsically disordered protein condensate-modified surface for mitigation of biofouling and foreign body response. J. Am. Chem. Soc. 144 (27), 12147–12157. 10.1021/jacs.2c02677 35767424

[B15] Charville G. W. Hetrick E. M. Geer C. B. Schoenfisch M. H. (2008). Reduced bacterial adhesion to fibrinogen-coated substrates via nitric oxide release. Biomaterials 29 (30), 4039–4044. 10.1016/j.biomaterials.2008.07.005 18657857 PMC2582185

[B16] Chen H. Liu C. Chen D. Madrid K. Peng S. Dong X. (2015). Bacteria-targeting conjugates based on antimicrobial peptide for bacteria diagnosis and therapy. Mol. Pharm. 12 (7), 2505–2516. 10.1021/acs.molpharmaceut.5b00053 26030231

[B17] Chopra I. (2007). The increasing use of silver-based products as antimicrobial agents: A useful development or a cause for concern? J. Antimicrob. Chemother. 59 (4), 587–590. 10.1093/jac/dkm006 17307768

[B18] Cochis A. Azzimonti B. Della Valle C. Chiesa R. Arciola C. R. Rimondini L. (2015). Biofilm formation on titanium implants counteracted by grafting gallium and silver ions. J. Biomed. Mat. Res. A 103 (3), 1176–1187. 10.1002/jbm.a.35270 25044610

[B19] Costa-Pinto A. R. Lemos A. L. Tavaria F. K. Pintado M. (2021). Chitosan and hydroxyapatite based biomaterials to circumvent periprosthetic joint infections. Mater. (Basel, Switz. 14 (4), 804. 10.3390/ma14040804 PMC7914941 33567675

[B20] Croes M. Bakhshandeh S. van Hengel I. A. J. Lietaert K. van Kessel K. P. M. Pouran B. (2018). Antibacterial and immunogenic behavior of silver coatings on additively manufactured porous titanium. Acta biomater. 81, 315–327. 10.1016/j.actbio.2018.09.051 30268917

[B21] Cunha M. T. Murça M. A. Nigro S. Klautau G. B. Salles M. J. C. (2018). *In vitro* antibacterial activity of bioactive glass S53P4 on multiresistant pathogens causing osteomyelitis and prosthetic joint infection. BMC Infect. Dis. 18 (1), 157. 10.1186/s12879-018-3069-x 29614973 PMC5883601

[B22] De Witte T. M. Fratila-Apachitei L. E. Zadpoor A. A. Peppas N. A. (2018). Bone tissue engineering via growth factor delivery: From scaffolds to complex matrices. Regen. Biomater. 5 (4), 197–211. 10.1093/rb/rby013 30094059 PMC6077800

[B23] Deb S. (2008). Orthopaedic bone cements || acrylic bone cement: Genesis and evolution, 167–182.

[B24] Dinda B. Dinda S. DasSharma S. Banik R. Chakraborty A. Dinda M. (2017). Therapeutic potentials of baicalin and its aglycone, baicalein against inflammatory disorders. Eur. J. Med. Chem. 131, 68–80. 10.1016/j.ejmech.2017.03.004 28288320

[B25] Dong J. Fang D. Zhang L. Shan Q. Huang Y. J. M. (2019). Gallium-doped titania nanotubes elicit anti-bacterial efficacy *in vivo* against *Escherichia coli* and *Staphylococcus aureus* biofilm. Mater. (Oxf). 5, 100209. 10.1016/j.mtla.2019.100209

[B26] Drago L. Boot W. Dimas K. Malizos K. Hänsch G. M. Stuyck J. (2014). Does implant coating with antibacterial-loaded hydrogel reduce bacterial colonization and biofilm formation *in vitro*? Clin. Orthop. Relat. Res. 472 (11), 3311–3323. 10.1007/s11999-014-3558-1 24622801 PMC4182393

[B27] Dunst S. Tomancak P. (2019). Imaging flies by fluorescence microscopy: Principles, technologies, and applications. Genetics 211 (1), 15–34. 10.1534/genetics.118.300227 30626639 PMC6325693

[B28] Engesæter L. B. Dale H. Schrama J. C. Hallan G. Lie S. A. (2011). Surgical procedures in the treatment of 784 infected THAs reported to the Norwegian Arthroplasty Register. Acta Orthop. 82 (5), 530–537. 10.3109/17453674.2011.623572 21992085 PMC3242948

[B29] Fang M. Adhikari R. Bi J. Mazi W. Dorh N. Wang J. (2017). Fluorescent probes for sensitive and selective detection of pH changes in live cells in visible and near-infrared channels. J. Mat. Chem. B 5 (48), 9579–9590. 10.1039/c7tb02583a PMC5875989 29607047

[B30] Feng Q. L. Wu J. Chen G. Q. Cui F. Z. Kim T. N. Kim J. O. (2000). A mechanistic study of the antibacterial effect of silver ions on *Escherichia coli* and *Staphylococcus aureus* . J. Biomed. Mat. Res. 52 (4), 662–668. 10.1002/1097-4636(20001215)52:4<662::aid-jbm10>3.0.co;2-3 11033548

[B31] Feng X. Lv F. Liu L. Tang H. Xing C. Yang Q. (2010). Conjugated polymer nanoparticles for drug delivery and imaging. ACS Appl. Mat. Interfaces 2 (8), 2429–2435. 10.1021/am100435k 20695494

[B32] Ferraris S. Miola M. Cochis A. Azzimonti B. Rimondini L. Prenesti E. (2017). *In situ* reduction of antibacterial silver ions to metallic silver nanoparticles on bioactive glasses functionalized with polyphenols. Appl. Surf. Sci. 396, 461–470. 10.1016/j.apsusc.2016.10.177

[B33] Foster A. L. Boot W. Stenger V. D'Este M. Jaiprakash A. Eglin D. (2021). Single-stage revision of MRSA orthopedic device-related infection in sheep with an antibiotic-loaded hydrogel. J. Orthop. Res. 39 (2), 438–448. 10.1002/jor.24949 33305875

[B34] Fu J. Ji J. Fan D. Shen J. (2006). Construction of antibacterial multilayer films containing nanosilver via layer-by-layer assembly of heparin and chitosan-silver ions complex. J. Biomed. Mat. Res. A 79 (3), 665–674. 10.1002/jbm.a.30819 16832825

[B35] Geredew Kifelew L. Mitchell J. G. Speck P. (2019). Mini-review: Efficacy of lytic bacteriophages on multispecies biofilms. Biofouling 35 (4), 472–481. 10.1080/08927014.2019.1613525 31144513

[B36] Guo Z. Park S. Yoon J. Shin I. (2014). Recent progress in the development of near-infrared fluorescent probes for bioimaging applications. Chem. Soc. Rev. 43 (1), 16–29. 10.1039/c3cs60271k 24052190

[B37] Hall-Stoodley L. Costerton J. W. Stoodley P. (2004). Bacterial biofilms: From the natural environment to infectious diseases. Nat. Rev. Microbiol. 2 (2), 95–108. 10.1038/nrmicro821 15040259

[B38] Hendriks J. G. van Horn J. R. van der Mei H. C. Busscher H. J. (2004). Backgrounds of antibiotic-loaded bone cement and prosthesis-related infection. Biomaterials 25 (3), 545–556. 10.1016/s0142-9612(03)00554-4 14585704

[B39] Hetrick E. M. Schoenfisch M. H. (2006). Reducing implant-related infections: Active release strategies. Chem. Soc. Rev. 35 (9), 780–789. 10.1039/b515219b 16936926

[B40] Hijazi S. Visaggio D. Pirolo M. Frangipani E. Bernstein L. Visca P. (2018). Antimicrobial activity of gallium compounds on ESKAPE pathogens. Front. Cell. Infect. Microbiol. 8, 316. 10.3389/fcimb.2018.00316 30250828 PMC6139391

[B41] Hoang Thi T. T. Lee Y. Le Thi P. Park K. D. (2018). Nitric oxide-releasing injectable hydrogels with high antibacterial activity through *in situ* formation of peroxynitrite. Acta biomater. 67, 66–78. 10.1016/j.actbio.2017.12.005 29269330

[B42] Høiby N. Bjarnsholt T. Givskov M. Molin S. Ciofu O. (2010). Antibiotic resistance of bacterial biofilms. Int. J. Antimicrob. agents 35 (4), 322–332. 10.1016/j.ijantimicag.2009.12.011 20149602

[B43] Honda M. Matsumoto M. Aizawa M. (2020). Potential application of protamine for antimicrobial biomaterials in bone tissue engineering. Int. J. Mol. Sci. 21 (12), 4368. 10.3390/ijms21124368 32575446 PMC7352774

[B44] Hussain S. Joo J. Kang J. Kim B. Braun G. B. She Z. G. (2018). Antibiotic-loaded nanoparticles targeted to the site of infection enhance antibacterial efficacy. Nat. Biomed. Eng. 2 (2), 95–103. 10.1038/s41551-017-0187-5 29955439 PMC6015743

[B45] Innes M. B. Atwater A. R. (2020). Orthopedic implant hypersensitivity reactions: Concepts and controversies. Dermatol. Clin. 38 (3), 361–369. 10.1016/j.det.2020.02.005 32475514

[B46] Inzana J. A. Schwarz E. M. Kates S. L. Awad H. A. (2016). Biomaterials approaches to treating implant-associated osteomyelitis. Biomaterials 81, 58–71. 10.1016/j.biomaterials.2015.12.012 26724454 PMC4745119

[B47] Ishihara M. Kishimoto S. Takikawa M. Hattori H. Nakamura S. Shimizu M. (2015). Biomedical application of low molecular weight heparin/protamine nano/micro particles as cell- and growth factor-carriers and coating matrix. Int. J. Mol. Sci. 16 (5), 11785–11803. 10.3390/ijms160511785 26006248 PMC4463730

[B48] Izakovicova P. Borens O. Trampuz A. (2019). Periprosthetic joint infection: Current concepts and outlook. EFORT open Rev. 4 (7), 482–494. 10.1302/2058-5241.4.180092 31423332 PMC6667982

[B49] Jin G. Mao D. Cai P. Liu R. Tomczak N. Liu J. (2015). Conjugated polymer nanodots as ultrastable long‐term trackers to understand mesenchymal stem cell therapy in skin regeneration. Adv. Funct. Mat. 25 (27), 4263–4273. 10.1002/adfm.201501081

[B50] Johnson C. T. Wroe J. A. Agarwal R. Martin K. E. Guldberg R. E. Donlan R. M. (2018). Hydrogel delivery of lysostaphin eliminates orthopedic implant infection by *Staphylococcus aureus* and supports fracture healing. Proc. Natl. Acad. Sci. U. S. A. 115 (22), E4960-E4969–e9. 10.1073/pnas.1801013115 29760099 PMC5984524

[B51] Jolivet-Gougeon A. Bonnaure-Mallet M. (2014). Biofilms as a mechanism of bacterial resistance. Drug Discov. Today Technol. 11, 49–56. 10.1016/j.ddtec.2014.02.003 24847653

[B52] Jones J. R. (2013). Review of bioactive glass: From Hench to hybrids. Acta biomater. 9 (1), 4457–4486. 10.1016/j.actbio.2012.08.023 22922331

[B53] Jung S. W. Oh S. H. Lee I. S. Byun J. H. Lee J. H. (2019). *In situ* gelling hydrogel with anti-bacterial activity and bone healing property for treatment of osteomyelitis. Tissue Eng. Regen. Med. 16 (5), 479–490. 10.1007/s13770-019-00206-x 31624703 PMC6778575

[B54] Kapadia B. H. Berg R. A. Daley J. A. Fritz J. Bhave A. Mont M. A. (2016). Periprosthetic joint infection. Lancet 387 (10016), 386–394. 10.1016/s0140-6736(14)61798-0 26135702

[B55] Kasugai S. Fujisawa R. Waki Y. Miyamoto K. Ohya K. (2000). Selective drug delivery system to bone: Small peptide (Asp)6 conjugation. J. Bone Min. Res. 15 (5), 936–943. 10.1359/jbmr.2000.15.5.936 10804024

[B56] Kazemi M. Ahangarani S. Esmailian M. Shanaghi A. (2020). Investigation on the corrosion behavior and biocompatibility of Ti-6Al-4V implant coated with HA/TiN dual layer for medical applications. Surf. Coatings Technol. 397, 126044. 10.1016/j.surfcoat.2020.126044

[B57] Kelly P. J. Li H. Benson P. S. Whitehead K. A. Verran J. Arnell R. D. (2010). Comparison of the tribological and antimicrobial properties of CrN/Ag, ZrN/Ag, TiN/Ag, and TiN/Cu nanocomposite coatings. Surf. Coatings Technol. 205 (5), 1606–1610. 10.1016/j.surfcoat.2010.07.029

[B58] Kumar J. K. (2008). Lysostaphin: An antistaphylococcal agent. Appl. Microbiol. Biotechnol. 80 (4), 555–561. 10.1007/s00253-008-1579-y 18607587

[B59] Kurtuldu F. Mutlu N. Boccaccini A. R. Galusek D. (2022). Gallium containing bioactive materials: A review of anticancer, antibacterial, and osteogenic properties. Bioact. Mater. 17, 125–146. 10.1016/j.bioactmat.2021.12.034 35386441 PMC8964984

[B60] Lee S. Kim S. Park J. Lee J. Y. (2020). Universal surface modification using dopamine-hyaluronic acid conjugates for anti-biofouling. Int. J. Biol. Macromol. 151, 1314–1321. 10.1016/j.ijbiomac.2019.10.177 31751701

[B61] Lenski M. Scherer M. A. (2015). Diagnostic potential of inflammatory markers in septic arthritis and periprosthetic joint infections: A clinical study with 719 patients. Infect. Dis. Lond. Engl. 47 (6), 399–409. 10.3109/00365548.2015.1006674 25746606

[B62] Levack A. E. Cyphert E. L. Bostrom M. P. Hernandez C. J. von Recum H. A. Carli A. V. (2018). Current options and emerging biomaterials for periprosthetic joint infection. Curr. Rheumatol. Rep. 20 (6), 33. 10.1007/s11926-018-0742-4 29713837 PMC6321987

[B63] Li D. Lv P. Fan L. Huang Y. Yang F. Mei X. (2017). The immobilization of antibiotic-loaded polymeric coatings on osteoarticular Ti implants for the prevention of bone infections. Biomater. Sci. 5 (11), 2337–2346. 10.1039/c7bm00693d 29034380

[B64] Li Y. Liu X. Li B. Zheng Y. Han Y. Chen D. F. (2020). Near-infrared light triggered phototherapy and immunotherapy for elimination of methicillin-resistant *Staphylococcus aureus* biofilm infection on bone implant. ACS Nano 14 (7), 8157–8170. 10.1021/acsnano.0c01486 32585104

[B65] Luo S. H. Xiao W. Wei X. J. Jia W. T. Zhang C. Q. Huang W. H. (2010). *In vitro* evaluation of cytotoxicity of silver-containing borate bioactive glass. J. Biomed. Mat. Res. 95 (2), 441–448. 10.1002/jbm.b.31735 20878930

[B66] McConoughey S. J. Howlin R. Granger J. F. Manring M. M. Calhoun J. H. Shirtliff M. (2014). Biofilms in periprosthetic orthopedic infections. Future Microbiol. 9 (8), 987–1007. 10.2217/fmb.14.64 25302955 PMC4407677

[B67] Miola M. Verné E. (2016). Bioactive and antibacterial glass powders doped with copper by ion-exchange in aqueous solutions. Mater. (Basel, Switz. 9 (6), 405. 10.3390/ma9060405 PMC5456756 28773530

[B68] Mohamed M. F. Hamed M. I. Panitch A. Seleem M. N. (2014). Targeting methicillin-resistant *Staphylococcus aureus* with short salt-resistant synthetic peptides. Antimicrob. Agents Chemother. 58 (7), 4113–4122. 10.1128/aac.02578-14 24798285 PMC4068565

[B69] Nemeno J. G. Lee S. Yang W. Lee K. M. Lee J. I. (2014). Applications and implications of heparin and protamine in tissue engineering and regenerative medicine. BioMed Res. Int. 2014, 1–10. 10.1155/2014/936196 PMC4065694 24995338

[B70] Nie B. Huo S. Qu X. Guo J. Liu X. Hong Q. (2022). Bone infection site targeting nanoparticle-antibiotics delivery vehicle to enhance treatment efficacy of orthopedic implant related infection. Bioact. Mater. 16, 134–148. 10.1016/j.bioactmat.2022.02.003 35386313 PMC8958424

[B71] Norouz Dizaji A. Ding D. Kutsal T. Turk M. Kong D. Piskin E. (2020). *In vivo* imaging/detection of MRSA bacterial infections in mice using fluorescence labelled polymeric nanoparticles carrying vancomycin as the targeting agent. J. biomaterials Sci. Polym. Ed. 31 (3), 293–309. 10.1080/09205063.2019.1692631 31762403

[B72] Palierse E. Hélary C. Krafft J-M. Génois I. Masse S. Laurent G. (2021). Baicalein-modified hydroxyapatite nanoparticles and coatings with antibacterial and antioxidant properties. Mater. Sci. Eng. C 118, 111537. 10.1016/j.msec.2020.111537 33255090

[B73] Palierse E. Hélary C. Krafft J. M. Génois I. Masse S. Laurent G. (2021). Baicalein-modified hydroxyapatite nanoparticles and coatings with antibacterial and antioxidant properties. Mater. Sci. Eng. C 118, 111537. 10.1016/j.msec.2020.111537 33255090

[B74] Park S. Y. Kim I. S. (2013). Identification of macrophage genes responsive to extracellular acidification. Inflamm. Res. 62 (4), 399–406. 10.1007/s00011-013-0591-6 23417272

[B75] Patron R. L. Climo M. W. Goldstein B. P. Archer G. L. (1999). Lysostaphin treatment of experimental aortic valve endocarditis caused by a *Staphylococcus aureus* isolate with reduced susceptibility to vancomycin. Antimicrob. Agents Chemother. 43 (7), 1754–1755. 10.1128/aac.43.7.1754 10390235 PMC89356

[B76] Petrovic Fabijan A. Lin R. C. Y. Ho J. Maddocks S. Ben Zakour N. L. Iredell J. R. (2020). Safety of bacteriophage therapy in severe *Staphylococcus aureus* infection. Nat. Microbiol. 5 (3), 465–472. 10.1038/s41564-019-0634-z 32066959

[B77] Pourshahrestani S. Zeimaran E. Kadri N. A. Gargiulo N. Jindal H. M. Naveen S. V. (2017). Potency and cytotoxicity of a novel gallium-containing mesoporous bioactive glass/chitosan composite scaffold as hemostatic agents. ACS Appl. Mat. Interfaces 9 (37), 31381–31392. 10.1021/acsami.7b07769 28836753

[B78] Prabhu M. Kavitha K. Suriyaprabha R. Manivasakan P. Rajendran V. Kulandaivelu P. (2013). Preparation and characterization of silver-doped nanobioactive glass particles and their <I&gt;*in vitro*&lt;/I&gt; behaviour for biomedical applications. J. Nanosci. Nanotechnol. 13 (8), 5327–5339. 10.1166/jnn.2013.7474 23882760

[B79] Premkumar A. Kolin D. A. Farley K. X. Wilson J. M. McLawhorn A. S. Cross M. B. (2021). Projected economic burden of periprosthetic joint infection of the hip and knee in the United States. J. arthroplasty 36 (5), 1484–1489.e3. e3. 10.1016/j.arth.2020.12.005 33422392

[B80] Rahaman M. N. Bal B. S. Huang W. (2014). Review: Emerging developments in the use of bioactive glasses for treating infected prosthetic joints. Mater. Sci. Eng. C 41, 224–231. 10.1016/j.msec.2014.04.055 24907755

[B81] Rahaman M. N. Bal B. S. Huang W. (2014). Review: Emerging developments in the use of bioactive glasses for treating infected prosthetic joints. Mater. Sci. Eng. C 41, 224–231. 10.1016/j.msec.2014.04.055 24907755

[B82] Rahaman M. N. Day D. E. Sonny Bal B. Fu Q. Jung S. B. Bonewald L. F. (2011). Bioactive glass in tissue engineering. Acta biomater. 7 (6), 2355–2373. 10.1016/j.actbio.2011.03.016 21421084 PMC3085647

[B83] Rameshbabu N. Sampath Kumar T. S. Prabhakar T. G. Sastry V. S. Murty K. V. Prasad Rao K. (2007). Antibacterial nanosized silver substituted hydroxyapatite: Synthesis and characterization. J. Biomed. Mat. Res. A 80 (3), 581–591. 10.1002/jbm.a.30958 17031822

[B84] Ribeiro M. Monteiro F. J. Ferraz M. P. (2012). Infection of orthopedic implants with emphasis on bacterial adhesion process and techniques used in studying bacterial-material interactions. Biomatter 2 (4), 176–194. 10.4161/biom.22905 23507884 PMC3568104

[B85] Rimondini L. Della Valle C. Cochis A. Azzimonti B. Chiesa R. (Editors) (2014). “The biofilm formation onto implants and prosthetic materials may be contrasted using Gallium (3+),” Key engineering materials (Trans Tech Publ).

[B86] Rivera L. R. Cochis A. Biser S. Canciani E. Ferraris S. Rimondini L. (2021). Antibacterial, pro-angiogenic and pro-osteointegrative zein-bioactive glass/copper based coatings for implantable stainless steel aimed at bone healing. Bioact. Mater. 6 (5), 1479–1490. 10.1016/j.bioactmat.2020.11.001 33251384 PMC7674162

[B87] Roca I. Akova M. Baquero F. Carlet J. Cavaleri M. Coenen S. (2015). The global threat of antimicrobial resistance: Science for intervention. New microbes new Infect. 6, 22–29. 10.1016/j.nmni.2015.02.007 26029375 PMC4446399

[B88] Rodríguez-Pardo D. Pigrau C. Corona P. S. Almirante B. (2015). An update on surgical and antimicrobial therapy for acute periprosthetic joint infection: New challenges for the present and the future. Expert Rev. Anti-infective Ther. 13 (2), 249–265. 10.1586/14787210.2015.999669 25578886

[B89] Ryan J. Jacob P. Lee A. Gagnon Z. Pavel I. E. (2022). “Biodistribution and toxicity of antimicrobial ionic silver (Ag(+)) and silver nanoparticle (AgNP(+)) species after oral exposure,” in Sprague-Dawley rats, 166, 113228.Food and chemical toxicology : an Int. J. Publ. Br. Industrial Biol. Res. Assoc. 10.1016/j.fct.2022.113228 35710031

[B90] Sabaté Del Río J. Henry O. Y. F. Jolly P. Ingber D. E. (2019). An antifouling coating that enables affinity-based electrochemical biosensing in complex biological fluids. Nat. Nanotechnol. 14 (12), 1143–1149. 10.1038/s41565-019-0566-z 31712665

[B91] Sanchez-Cano C. Carril M. (2020). Recent developments in the design of non-biofouling coatings for nanoparticles and surfaces. Int. J. Mol. Sci. 21 (3), 1007. 10.3390/ijms21031007 32028729 PMC7037411

[B92] Sánchez-López J. C. Abad M. D. Carvalho I. Escobar Galindo R. Benito N. Ribeiro S. (2012). Influence of silver content on the tribomechanical behavior on Ag-TiCN bioactive coatings. Surf. Coatings Technol. 206 (8), 2192–2198. 10.1016/j.surfcoat.2011.09.059

[B93] Schöttler S. Becker G. Winzen S. Steinbach T. Mohr K. Landfester K. (2016). Protein adsorption is required for stealth effect of poly(ethylene glycol)- and poly(phosphoester)-coated nanocarriers. Nat. Nanotechnol. 11 (4), 372–377. 10.1038/nnano.2015.330 26878141

[B94] Shimazaki T. Miyamoto H. Ando Y. Noda I. Yonekura Y. Kawano S. (2010). *In vivo* antibacterial and silver-releasing properties of novel thermal sprayed silver-containing hydroxyapatite coating. J. Biomed. Mat. Res. B Appl. Biomater. 92 (2), 386–389. 10.1002/jbm.b.31526 19904818

[B95] Slaughter B. V. Khurshid S. S. Fisher O. Z. Khademhosseini A. Peppas N. A. (2009). Hydrogels in regenerative medicine. Adv. Mat. 21 (32-33), 3307–3329. 10.1002/adma.200802106 PMC4494665 20882499

[B96] Smith B. R. Gambhir S. S. (2017). Nanomaterials for *in vivo* imaging. Chem. Rev. 117 (3), 901–986. 10.1021/acs.chemrev.6b00073 28045253

[B97] Stuart B. W. Grant C. A. Stan G. E. Popa A. C. Titman J. J. Grant D. M. (2018). Gallium incorporation into phosphate based glasses: Bulk and thin film properties. J. Mech. Behav. Biomed. Mater. 82, 371–382. 10.1016/j.jmbbm.2018.03.041 29656232

[B98] Ter Boo G. A. Arens D. Metsemakers W. J. Zeiter S. Richards R. G. Grijpma D. W. (2016). Injectable gentamicin-loaded thermo-responsive hyaluronic acid derivative prevents infection in a rabbit model. Acta biomater. 43, 185–194. 10.1016/j.actbio.2016.07.029 27435965

[B99] Valencia Zapata M. E. Mina Hernandez J. H. Grande Tovar C. D. Valencia Llano C. H. Diaz Escobar J. A. Vázquez-Lasa B. (2019). Novel bioactive and antibacterial acrylic bone cement nanocomposites modified with graphene oxide and chitosan. Int. J. Mol. Sci. 20 (12), 2938. 10.3390/ijms20122938 31208091 PMC6627441

[B100] Wang H. Maeda T. Miyazaki T. (2021). Preparation of bioactive and antibacterial PMMA-based bone cement by modification with quaternary ammonium and alkoxysilane. J. Biomater. Appl. 36 (2), 311–320. 10.1177/08853282211004413 33757363

[B101] Ward T. T. Steigbigel R. T. (1978). Acidosis of synovial fluid correlates with synovial fluid leukocytosis. Am. J. Med. 64 (6), 933–936. 10.1016/0002-9343(78)90446-1 26220

[B102] Wekwejt M. Chen S. Kaczmarek-Szczepańska B. Nadolska M. Łukowicz K. Pałubicka A. (2021). Nanosilver-loaded PMMA bone cement doped with different bioactive glasses - evaluation of cytocompatibility, antibacterial activity, and mechanical properties. Biomater. Sci. 9 (8), 3112–3126. 10.1039/d1bm00079a 33704333

[B103] Wijayaratna U. N. Kiridena S. D. Adams J. D. Behrend C. J. Anker J. N. J. A. F. M. (2021). Synovial fluid pH sensor for early detection of prosthetic hip infections. 10.1002/adfm.202104124 PMC9725744 36478668

[B104] Wroe J. A. Johnson C. T. García A. J. (2020). Bacteriophage delivering hydrogels reduce biofilm formation *in vitro* and infection *in vivo* . J. Biomed. Mat. Res. A 108 (1), 39–49. 10.1002/jbm.a.36790 PMC6880309 31443115

[B105] Wu J. A. Kusuma C. Mond J. J. Kokai-Kun J. F. (2003). Lysostaphin disrupts *Staphylococcus aureus* and Staphylococcus epidermidis biofilms on artificial surfaces. Antimicrob. Agents Chemother. 47 (11), 3407–3414. 10.1128/aac.47.11.3407-3414.2003 14576095 PMC253758

[B106] Xie Z. Liu X. Jia W. Zhang C. Huang W. Wang J. (2009). Treatment of osteomyelitis and repair of bone defect by degradable bioactive borate glass releasing vancomycin. J. Control. Release 139 (2), 118–126. 10.1016/j.jconrel.2009.06.012 19545593

[B107] Xu Z. Zhao X. Chen X. Chen Z. Xia Z. J. Ra (2017). Antimicrobial effect of gallium nitrate against bacteria encountered in burn wound infections. RSC Adv. 7 (82), 52266–52273. 10.1039/c7ra10265h

[B108] Yamaguchi S. Nath S. Sugawara Y. Divakarla K. Das T. Manos J. (2017). Two-in-One biointerfaces-antimicrobial and bioactive nanoporous gallium titanate layers for titanium implants. Nanomater. (Basel, Switz. 7 (8), 229. 10.3390/nano7080229 PMC5575711 28825641

[B109] Yamamuro T. Nakamura T. Oka M. J. B. (1995). Transmission electron microscopy observations at the interface of bone and four types of calcium phosphate ceramics with different calcium/phosphorus molar ratios. Biomaterials 16 (14), 1101–1107. 10.1016/0142-9612(95)98907-v 8519932

[B110] Yang T. Zelikin A. N. Chandrawati R. (2018). Progress and promise of nitric oxide-releasing platforms. Adv. Sci. (Weinh). 5 (6), 1701043. 10.1002/advs.201701043 29938181 PMC6010811

[B111] Zhang D. Munukka E. Leppaeranta O. Hupa L. Ylaenen H. Salonen J. (2006). Comparison of antibacterial effect of three bioactive glasses. Key Eng. Mat. 309-311 (1), 345–348. 10.4028/www.scientific.net/kem.309-311.345

[B112] Zhang S. Qu X. Jiao J. Tang H. Wang M. Wang Y. (2022). Felodipine enhances aminoglycosides efficacy against implant infections caused by methicillin-resistant *Staphylococcus aureus*, persisters and biofilms. Bioact. Mater. 14, 272–289. 10.1016/j.bioactmat.2021.11.019 35310349 PMC8897655

[B113] Zilberman M. Elsner J. J. (2008). Antibiotic-eluting medical devices for various applications. J. Control. Release 130 (3), 202–215. 10.1016/j.jconrel.2008.05.020 18687500

